# Risk factors associated with cassava brown streak disease dissemination through seed pathways in Eastern D.R. Congo

**DOI:** 10.3389/fpls.2022.803980

**Published:** 2022-07-22

**Authors:** Yves Kwibuka, Chantal Nyirakanani, Jean Pierre Bizimana, Espoir Bisimwa, Yves Brostaux, Ludivine Lassois, Herve Vanderschuren, Sebastien Massart

**Affiliations:** ^1^Plant Pathology Laboratory, TERRA-Gembloux Agro-Bio Tech, University of Liège, Gembloux, Belgium; ^2^Faculté des Sciences Agronomiques, Université Catholique de Bukavu, Bukavu, Democratic Republic of Congo; ^3^Plant Genetics Laboratory, TERRA-Gembloux Agro-Bio Tech, University of Liège, Gembloux, Belgium; ^4^Department of Research, Rwanda Agriculture and Animal Resources Development Board, Huye, Rwanda; ^5^Applied Statistics, Computer Science and Modeling Laboratory, TERRA-Gembloux Agro-Bio Tech, University of Liège, Gembloux, Belgium; ^6^Laboratory of Tropical Crop Improvement, Division of Crop Biotechnics, Department of Biosystems, KU Leuven, Leuven, Belgium

**Keywords:** viral diseases, epidemiology, seed system, risk factors, molecular diagnostic, cassava [*Manihot esculenta* (L.) Crantz]

## Abstract

Vegetatively propagated crops are particularly prone to disease dissemination through their seed systems. Strict phytosanitary measures are important to limit the impact of diseases as illustrated by the potato seed system in Europe. Cassava brown streak disease (CBSD) is a devastating disease caused by two viral species collectively named cassava brown streak viruses (CBSVs). CBSD can cause substantial root yield losses of up to 100% in the worst affected areas and is easily transmitted through stem cuttings. In Eastern and Central Africa, the epidemiology of CBSVs in the local socio-economical context of production remains poorly known while a better understanding would be an asset to properly manage the disease. This lack of information explains partially the limited efficiency of current regulatory schemes in increasing the availability of quality seed to smallholders and mitigating the spread of pests and diseases. This study surveyed the epidemiology of CBSVs in Uvira territory, Eastern D.R. Congo, and its drivers using a multivariate approach combining farmer’s interview, field observation, sampling and molecular detection of CBSVs. Investigation on the epidemiology of CBSD revealed that three clusters in the study area could be identified using five most significant factors: (i) symptoms incidence, (ii) number of whiteflies, (iii) types of foliar symptoms, (iv) cutting’s pathways and (v) plant age. Among the three clusters identified, one proved to be potentially interesting for seed multiplication activities since the disease pressure was the lowest. Through risk assessment, we also identified several key socio-economic determinants on disease epidemy: (i) factors related to farmer’s knowledge and awareness (knowledge of cassava pests and diseases, knowledge of management practices, support from extension services and management strategies applied), (ii) factors related to the geographical location of farmer’s fields (proximity to borders, proximity to town, distance to acquire cuttings), as well as (iii) the pathways used to acquire cuttings.

## Introduction

Cassava (*Manihot esculenta* Crantz) is the tenth most important crop in the world in terms of global annual production (303 Million tons) (FAO, 2020). In Africa, its importance in the livelihood of populations has long been demonstrated: it is ranked the first most important food crop in terms of global annual production (192 Million tons in 2019), the first in terms of source of food (76 Million tons) and the fourth in terms of source of calories (167 Kcal/person/day) after wheat, maize and rice (FAO, 2021a). It is the most important food crop (occupying approximately 40% of agricultural land dedicated to food crops) and the largest non-cereal carbohydrate source for more than 70% of people in D.R. Congo (Mahungu et al., 2014). According to statistics from 2019, this country is ranked the second in Africa in term of production (40 Million tons) after Nigeria (59 Million tons; FAO, 2021a).

One of the crucial factors to increase agricultural productivity is the planting material: in this paper stem cuttings used for the propagation of cassava are referred to as *seed* (McEwan et al., 2021). Farmers often use different approaches to obtain seeds of a crop. Because of their variability and local specificity to needs and preferences, local approaches (e.g., household stocks, markets, and social exchange networks) provide usually most of the seeds that small farmers use (Sperling and Cooper, 2003). These local approaches are the components of the local seed system for which common figures suggested they would provide between 80 and 90% of the planting material to farmers in Sub-Saharan Africa (Danagro, 1988; Rabobank, 1994).

Seed systems of root, tuber, banana and other vegetatively propagated crops (VPC) are predominantly informal or managed at local levels by farmers themselves without major public or private sector involvement in the production, supply, or quality control of planting materials. The quality of seeds is often signaled through trust and reputation while the vegetative mode of seed multiplication increase the risk of pathogens, including viruses, building up over multiple cycles of propagation (Campo et al., 2011; Jarvis et al., 2012; Almekinders et al., 2019; McEwan et al., 2021). In practice, most farmers in low-income countries save seed from the previous season for replanting (Devaux et al., 2014).

Cassava cultivation is suffering significant losses due to biotic stresses (Vanderschuren and Rey, 2017). Lozano and Booth (1974); Reddy (2015); and Kwibuka et al. (2021) among which two viral diseases, cassava brown streak disease (CBSD) and cassava mosaic disease (CMD) are of major economic importance in sub-Saharan Africa (Vanderschuren and Rey, 2017). In Africa, previous estimates indicated overall incidences of 50 to 60% with estimated annual losses of $1.2–2.4 billion for CMD (Thresh et al., 1997; Legg et al., 2006), while annual economic losses of up to US $ 726 million were associated to CBSD with incidences of up to 100% being recorded (Maruthi, 2015). The situation is far from being controlled as future CBSD pressure is projected to increase by at least 2% in D.R. Congo by 2030 (Jarvis et al., 2012).

CBSD is associated with two Ipomovirus species (collectively named CBSVs), named cassava brown streak virus (CBSV) and Uganda cassava brown streak virus (UCBV) (Lozano and Booth, 1974; Bisimwa, 2012; Reddy, 2015). This disease is considered as endemic to low-altitude and coastal zones of Kenya, Tanzania, and Mozambique (Storey, 1936; Nichols, 1950). New outbreaks into areas 1,200 meters above sea level of Kenya, Uganda, Malawi and D.R. Congo indicated later a significant shift in its epidemiology and a westward progression to areas previously not at risk (Alicai et al., 2007; Mulimbi et al., 2012; Tomlinson et al., 2018).

Recently, several studies tried to elucidate the components of pathogens and arthropod pest invasion risk. Factors such as climatic conditions (Kroschel et al., 2016), structure of trade routes (Bebber et al., 2014) as well as habitat (cropland) connectivity (Xing et al., 2020) have been reported to play a major role in pest and disease dissemination. However, these studies have addressed these aspects from a global point of view and specific analysis of cassava crop is still missing. Additionally, network studies (Shaw and Pautasso, 2014) improved the understanding of the pathways of pathogens spreads. Such study can facilitate the reduction of the disease inoculum flow in exchanged plant materials by identifying likely ways to find the best sites to monitor as warning sites. The risk that pathogens can move through particular pathways of a seed system network is a key component of disease risk, along with other risk factors such as potential transmission by vectors or wind dispersal (Buddenhagen et al., 2017).

Pathways (sources) by which farmers obtain cassava seeds (cuttings) are of key importance in the mitigation of plant diseases. Accumulation and spread of viruses in planting material of vegetatively propagated crops provide the primary inoculum on field and are a key factor in the development of disease epidemics. This impact is particularly important when cuttings of susceptible cultivars used by farmers come from pathways without any sanitation measure (Frangoie et al., 2019). Therefore, understanding in a timely manner which cutting pathway/source has a high risk of pathogen dissemination in a seed network is a milestone for mitigation measures to be implemented.

Previous research has identified four factors impacting the spread of cassava viral diseases in most of affected areas in Africa: education and access to information, lack of plant health and extension services, weak access to improved varieties (FAO, 2021b) and social factors, exemplified by kinship systems (Delêtre et al., 2021).

The international Plant Protection Convention (IPPC) has proposed a standardized approach for assessing and managing the phytosanitary risk of a pest and/or of a pathway [Pest Risk Assessment (PRA)] (IPPC, 2021). This study focuses on a component of PRA related to the probability of spread of an established pest (CBSV) through pathways used by farmers to access planting material of a vegetatively propagated crop. It aimed at (i) establishing the epidemic profile of CBSD in Uvira territory by identifying the viruses occurring and describing their spatial distribution and (ii) identifying multidisciplinary factors underpinning the spread of CBSD and that can be useful in generating necessary knowledge for seed quality assurance, clean seed use and ultimately better CBSD control.

## Materials and methods

### Conceptual framework

**Figure 1:**
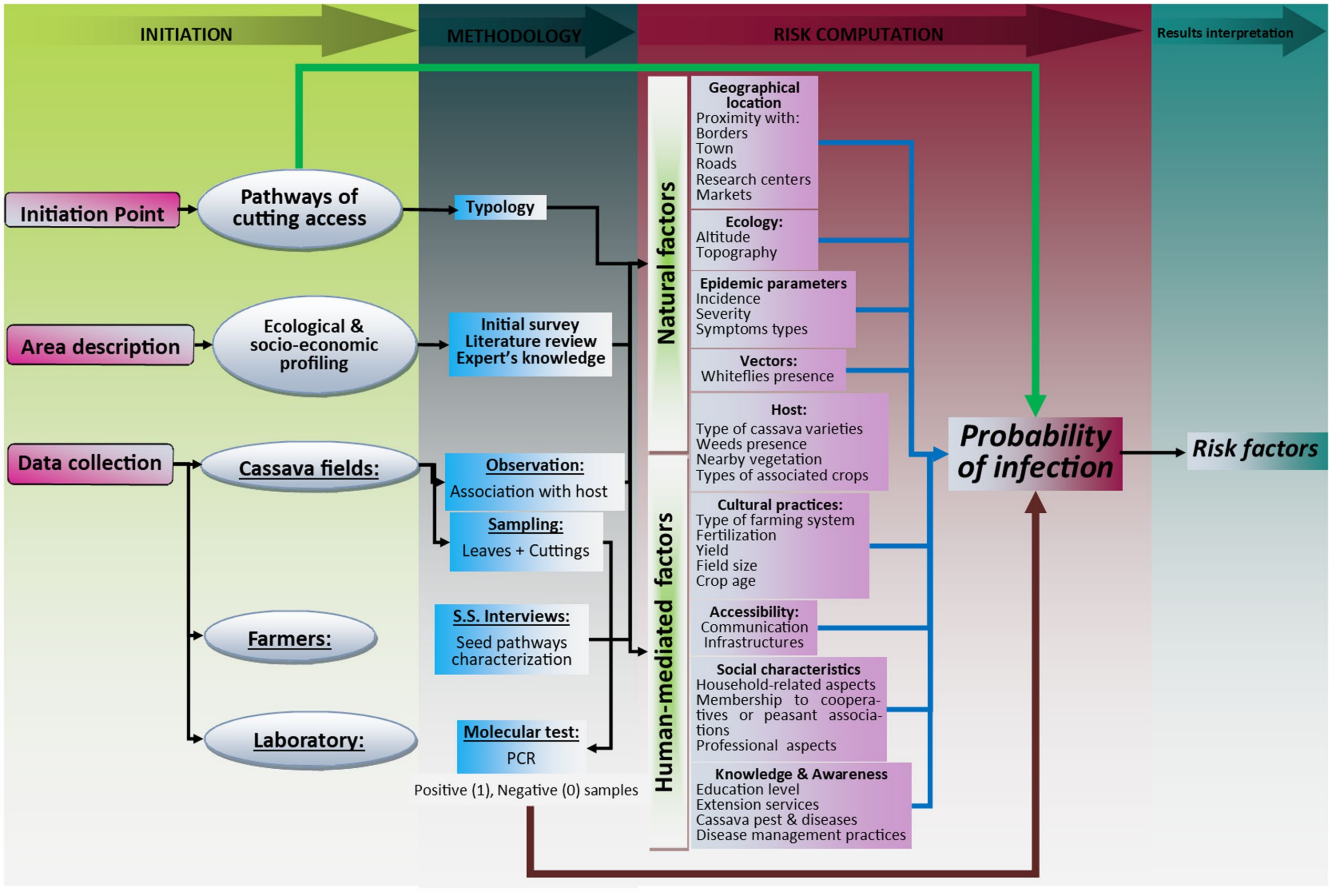
Illustration of the conceptual framework adopted in this study.

Two sets of factors that can play a role in the outcome of CBSV infection were included within the model adopted in this study (Figure 1). The first set (considered here as independent variables) is related to cutting pathways (diversity and characteristics) used by farmers and the second set (considered as intermediate variables) is related to human-mediated (Bebber et al., 2014; Delêtre et al., 2021; FAO, 2021b) and environmental factors (Kroschel et al., 2016; Buddenhagen et al., 2017; Xing et al., 2020). Risk factors are those associated positively and significantly to the increase of the number of diseased plants and therefore to a significant increase in the probability of CBSD infection.

Among other factors, human-mediated factors included (i) farmer’s demographic factors (sex, age, marital status, size of household, household headship and relation to the head of household), (ii) knowledge based factors (education level, access to training, experience in cassava farming, knowledge of cassava pests and diseases, knowledge of management practices against cassava pests, access to extension services, membership to cooperatives), (iii) economic-based factors (land size, land ownership, livestock ownership, access to inputs, main sources of income, labor type and availability) and, (iv) farming practices (type of farming system, field hygiene and rotation, planting and harvesting periods, use of disease-free planting material, use of resistant/improved varieties). Environmental factors included the (i) geographical location of fields (proximity with country borders, roads, main cities, markets, research centers, crop diversity around cassava field etc.), (ii) ecological factors (altitude, topography) and (iii) epidemiology (incidence, severity, type of symptoms, vectors, hosts etc.) etc.

### Study area

**Figure 2:**
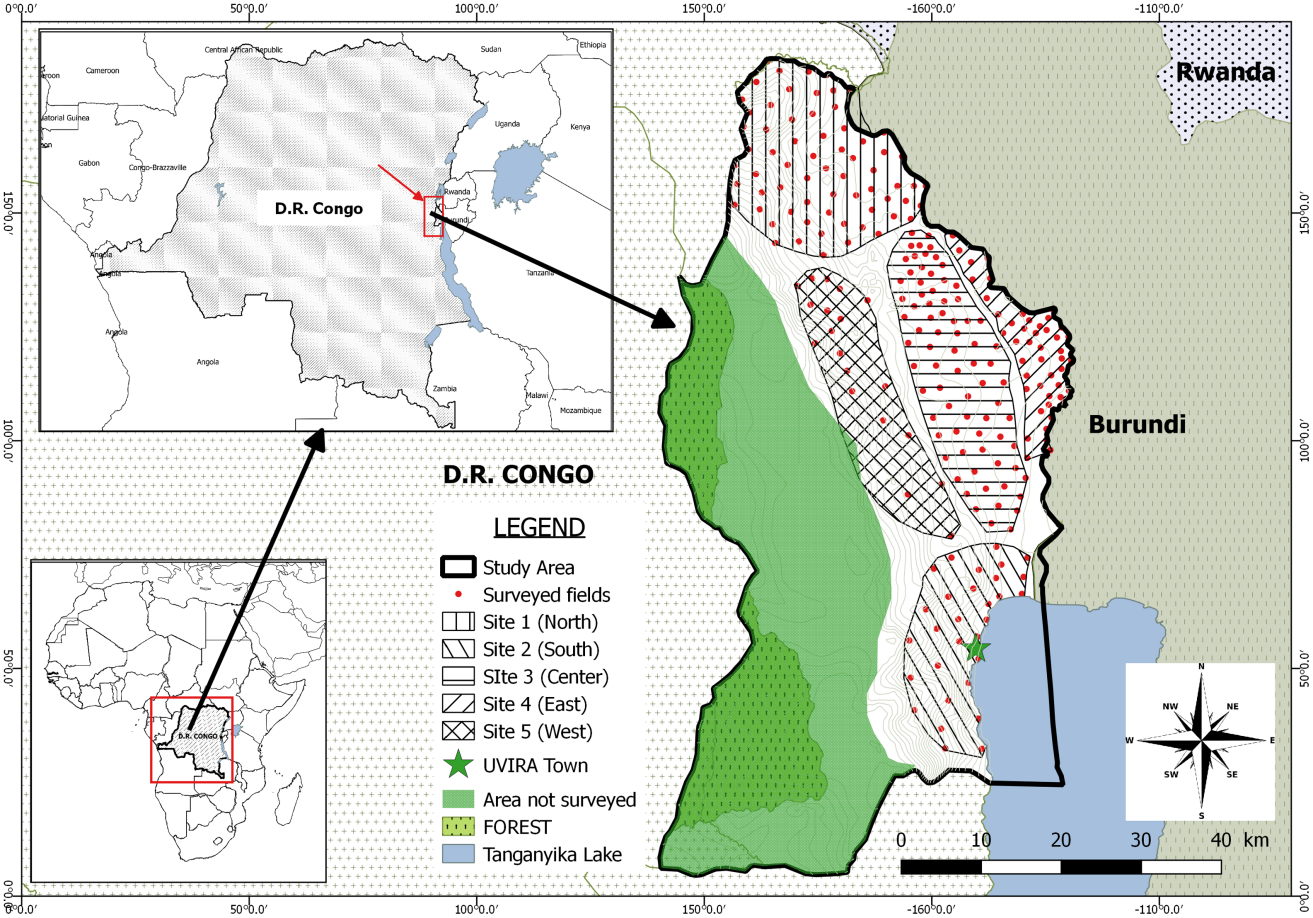
Geographic map of the study area showing subdivision into 5 sites. Red dots represent surveyed fields.

This study was conducted in Uvira territory, one of the eight administrative entities composing the South Kivu province in the Eastern D.R. Congo (Figure 2). This territory is composed of two ecological regions located in tropical zone of low altitude (climate type AW_1-3_, altitude lower than 1,000 m, rainfall <1,300 mm/year and annual mean temperature > 24°C) and in tropical zone of mild and high altitude (climate type Am, altitude between 1,000 and 1800 m, rainfall <1,600 mm/year and annual mean temperature < 23°C) respectively (Bisimwa, 2012). This territory is ranked among the top cassava producers of the province (IPA, 2020).

### Farmer and field selection

The design adopted in this study consisted into a multistage approach to select farmers and fields to survey. In the first stage, a purposive sampling strategy was used to select villages to be surveyed. In fact, from an initial sampling frame constituted by the total number of villages in the territory (226), sixty-five villages were retained after discarding villages where cassava productivity was marginal. A preliminary survey as well as literature review allowed to document the main socio-economic and ecological parameters susceptible to play a role in the local epidemiology of CBSD and impacting the way farmers access cuttings. However, some parameters were voluntary ignored because considered as potentially leading to conflicts or inappropriate by the local administrators (village chiefs, local agricultural officers). According to these harmonized parameters (Table 1), the 65 villages were clustered into 5 study sites: the North (site1), the South (site 2), the center (site 3), the East (site 4) and the West (site 5; Figure 2). Using official production statistics from 2019 (IPA, 2020) related to the amount of cassava produced, a typology of villages was established and consisted of villages with high intensity of production (annual cassava production >1,000 tons) as well as those of low intensity (annual cassava production <1,000 tons).

**Table 1:** Characteristics of the five sites.

Denomination	Location	Main villages	Characteristics
Site 1	North	Kamanyola, Luvungi, Bwegera, Kiringye, Katogota, Ndolera, Lubarika	Share border with both Rwandan and Burundian Republics
			Distant from the administrative seat of the territory (Uvira, 70 km)
			The topography is mixed (plain and mountains)
Site 2	South	Rutemba, Muhungu, Kavimvira, Kalungwe, Sango	Close to the Uvira city (the administrative main town of the territory)
			The topography is dominated by mountains
Site 3	Center	Kitemesho, Luberizi, Mutarule, Nyakabere, Sange, Runingu	Most of villages are close to the main national road NR1
			Located entirely in the low altitude zone (uniform topography)
			Villages are easily accessible
Site 4	East	Rwenena, Ndunda, Rusabagi, Sasira, Kigurwe, Rurimbi, Ruzia, Mwaba	Located on the border close to Republic of Burundi
			Distant from the national road 1 crossing the territory.
			Located entirely in the low altitude zone (uniform topography).
Site 5	West	Rubanga, Langala, Lemera, Mushegereza, Mulenge, Lusheke, Mugaja, Kanga	Located completely in mid or high altitude and dominated by mountains
			The area is poorly accessible
			Population density is lower compared to other sites
			Most agro-ecological characteristics differs from other sites

In the second stage, a simple random sampling strategy was used to select farmers and fields in each village. Four farmers and their corresponding fields were surveyed within each village belonging to the first category while at least 2 fields surveyed within villages belonging to the second category. These numbers of fields were determined according to the time and resources assigned to this work. Random numbers generated in Microsoft excel and assigned to the list of cassava producers for each village allowed to select fields to visit. These fields were identified under the lead of local agricultural officers. Fields were distant of at least 2 km and grown with cassava plants of more than 6 months old. An official authorization letter to conduct this study was delivered by the authorities of the Université Catholique de Bukavu and served as the official communication to local administrative chiefs who, in return granted authorization to undertake the study in entities under their responsibility.

The survey consisted into semi-structured interviews using mobile-recorded questionnaires using the Open Data Kit platform (Hartung et al., 2010) and was conducted directly on field site (except for some cases when the farmer could not be present on field). This strategy allowed to perform field inspections directly after interviews and to record information regarding the epidemiology of CBSD. Before starting interviews and performing sampling, a voluntary agreement of farmers was required through an explanation of the purposes of this study. In return, the farmer had to give an oral consent and additional explanations regarding confidentiality of collected data were also provided if required by the farmer.

### Epidemic survey

Epidemic parameters that were observed and recorded consisted in symptoms incidence, symptom types, symptoms severity on leaves and stems as well as whitefly number. These observations were conducted on systematically selected plants (see below for further details; Munthali, 1992; Hillocks et al., 1999; Hillocks and Thresh, 2000; Hillocks and Jennings, 2003; Rwegasira and Rey, 2012a).

CBSD symptoms are reported to be expressed as chloroses and necroses in leaves, stems, roots, and sometimes on green fruits. However these symptoms have been documented as being variable depending on the type of the cultivar infected (Hillocks and Jennings, 2003), the age of the plant (Hillocks et al., 1999) as well as the environmental conditions (Munthali, 1992). When a susceptible cultivar is contaminated through a cutting-derived infection, symptoms start soon after sprouting and consists in pronounced foliar and root symptoms. In leaves, chlorosis is associated with the main veins or rather in blotches unconnected to veins. These symptoms are prominent on mature lower leaves and completely absent on young and freshly expanded leaves (Rwegasira and Rey, 2012a). On stems, symptoms consist into brown, round or elongate streak-like lesions on the young green portion of infected stems. They starts as minor necrotic spots which fuse into bigger necrotic lesions culminating into shoot die-back as most of the tender portion of stem becomes necrotic (Hillocks and Jennings, 2003). Root symptoms consists into dried, brown necrotic lesions in the storage tissues and sometimes root constrictions. Sometimes, brown or black lesions on green fruits, and necrotic lesions in leaf scars are observed. When the infection become severe, these lesions develop to kill the dormant axillary buds, leading to a general shrinkage of the node and death of the internodal tissue, so that the branch dies from the tip to cause “dieback” (Hillocks and Thresh, 2000).

Symptoms incidence observation was conducted on 30 cassava plants encountered following diagonals and medians through the field (see below for the details; Hillocks, 2004; Rwegasira and Rey, 2012b). This parameter was then recorded as the number of plants that showed CBSD-like symptoms out of the 30 observed. Symptoms severity on aerial plant parts was assessed using the 5 levels scoring-scale from (Alicai et al., 2016). The number of whiteflies was counted on the top five youngest leaves of cassava plants selected for observation. The type of symptoms were categorized based on distribution of leaf chlorosis and stem lesions on the plant; systemic and on the whole plant (SW), systemic on leaf or stem parts but localized (SL), only on lower leaves (LL) (Alicai et al., 2016).

### Sample collection: Leaves and stem cuttings

Two rounds of inspection were undertaken in each cassava field of 6 months old to collect epidemiological data as well as leaf and stem cuttings: the first round consisted into symptoms observation and scoring while the second consisted into sample collection. In the first round, 30 plants were systematically selected and labelled in the field (see Supplementary Figure 1 for the clear illustration) in such a way that 5 plants had to be located on each diagonal (D1*_1_* to D1*_5_* for the first diagonal, D2*_1_* to D2*_5_* for the second diagonal), 5 plants on each median (M1*_1_* to M1*_5_* for the first median, M2*_1_* to M2*_5_* for the second median) as well as 10 plants on the outer borders of the field. Ten plants out of the 30 assessed for the symptoms presence in the first round were systematically selected for sample collection in the second round including 6 plants on both diagonals (D1*_1_*, D1*_3_*, D1*_5_*, D2*_1_*, D2*_3_*, D2*_5_*) and 4 plants on both medians (M1*_1_*, M1*_4_*, M2*_1_*, M2*_5_*). This strategy was applied in all of the 240 fields assessed. The figure presented in Supplementary Figure 1 illustrates clearly this process. From the top of the plant canopy downwards, 400–600 mg of the third fully expanded leaf on the major stem were collected, silica gel-dried and stored into sealed plastic tubes pending RNA extraction. Young leaves were preferred as content of polysaccharides and polyphenols, interfering with the molecular detection of the viruses, are lower (Shankar et al., 2010; Zhang and Stewart, 2016; Orek, 2018; Heikrujam et al., 2020). A total of 2,400 leaf samples were therefore collected from the 240 cassava fields of at least 6 months old. Two stem cuttings having at least 6 node buds were also sampled in the middle part of each selected plant using a pair of shear. The stem cuttings were labelled and stored pending re-plantation in an experimental field. A total of 480 stem cuttings were therefore collected.

### Molecular analysis

#### RNA extraction

The 10 separate tubes containing silica gel-dried leaf-tissues from unique fields were pooled (50 mg for each sample) to constitute 240 pooled samples that were shipped to Belgium. Total nucleic acid was extracted from each pooled sample (*n* = 240) using a modified CTAB protocol (Chang et al., 1993; Moreno et al., 2011).

Each pool were transferred into a thick-gauged plastic grinding bag and 2 ml of CTAB extraction buffer (2% CTAB, 2% PVP, 100 mM Tris–HCl pH 8.0, 25 mM EDTA, 2 M NaCl and 2% β-Mercaptoethanol added before use) was added and the leaf tissues were thoroughly grounded using a hand-held ball bearing sample grinder. 1 ml of the lysate was transferred to a 2 ml Eppendorf tube, homogenized and incubated at 60°C for 30 min with periodic vortexing at 10 min interval. 600 μl of chloroform: IAA (24:1) was added and the mix homogenized by inverting the tube. Phases were separated by centrifugation at maximum speed for 10 min at 4°C and the supernatant (upper aqueous phase) thoroughly transferred to a 1.5 ml Eppendorf tube. This Chloroform-IAA treatment was repeated once and 0.6 volumes of ice-cold Isopropanol (−20°C) was then added. Samples were allowed to stand for 2 h at −20°C then centrifuged for 30 min at 4°C to pellet the nucleic acid. Supernatant was removed and 0.5 ml of 70% Ethanol added to wash the pellet by centrifugation at 4°C for 5 min at maximum speed. Supernatant was removed, the pellet air-dried, re-suspended in 100 μl of TE buffer and stored on ice. Samples were DNase-treated using Amplification Grade DNASE I (Invitrogen^®^, United States) according to the manufacturer’s instructions and the quality of RNA tested using a Nanodrop 2000 spectrophotometer (Thermo Fisher Scientific, Waltham, MA, United States). Samples were aliquoted and stored at −80°C prior to testing.

#### CBSV and UCBSV detection by RT-PCR

All samples were tested for CBSV and UCBSV using a two-step RT-PCR assay. cDNA synthesis was carried out with Tetro^™^ reverse transcriptase (Meridian Bioscience^®^) according to manufacturer’s instructions. Random hexamers primers were used for generating the first strand cDNA. Amplification of cDNA was done using Mangotaq^™^ DNA polymerase (Meridian Bioscience^®^). Degenerated primer pair targeting the coat protein genes of both CBSV and UCBSV was used [CBSVs-F (5´-CCTCCATCWCATGCTATAGACA-3′) and CBSDD-R (5´-GGATATGGAGAAAGRKCTCC-3′)] (Anjanappa et al., 2016). These primer pair amplified a product of ~703 bp in the presence of CBSV and a product of ~800 bp in the presence of UCBSV. A 10 μl PCR mixture containing 5.8 μl nuclease free water, 2 μl PCR buffer (5X), 0.40 μl MgCl_2_ (50 mM), 0.20 μl dNTPs (10 mM), 0.20 μl of each primer (10 mM), 0.4 μl Mango taq DNA polymerase (Meridian Bioscience^®^) and 1.0 μl of cDNA. The temperature profile of PCR consisted of 95°C for 2 min followed by 30 cycles of 94°C (30 s), 56°C (30 s) and 72°C (50 s) for denaturation, annealing and extension, respectively. A final elongation of 72°C for 5 min was also included to terminate the amplification. The cassava *PP2A* gene was used as internal control gene in parallel reactions with the following primer pair (PP2A-F: 5´-TGCAAGGCTCACACTTTCATC-3′ and PP2A-R: 5´-CTGAGCGTAAAGCAGGGAAG-3′; Moreno et al., 2011). The *PP2A* primer pair generates an ~187-bp amplicon from cassava cDNA samples. PCR products were analyzed by electrophoresis in TAE buffer (1X) on a 1% agarose gel stained with Gel red^®^ (Biotium), visualized under UV light and photographed using a gel documentation system (E-Box CX5 Edge, Vilber/Fisher Biotech).

### Data analysis

Data collected from surveys were CSV-formatted and resulting files were loaded into R Software version 4.1.1 for analysis.

#### Descriptive statistics

Factors related to sites and clusters served as grouping factors to average the epidemic parameters (field symptom incidence, molecular detection incidence and severity score). The averaging of symptoms incidence and severity in cassava fields included both symptomatic and asymptomatic plants.

To get information about the CBSD incidence with regards to clusters, a generalized linear regression with logit link was applied (Agresti, 2002; Dodge, 2008), namely logit(p) = ln(p/(1-p)). The regression equation used was therefore written as:


$$logit\left(p\right)=\ln\left(p/\left(1-p\right)\right)=\beta_{0}+\beta_{1}*Cluster+Error$$

where *p* is the probability of the dependent variable [0; 1], *β*_n_ the regression coefficients, and “Cluster” an explanatory variable with 3 levels. The model parameters were estimated using the maximum likelihood method (Everitt, 2006), with Chi-squared test for significance, and the least-squared means comparison by the “lsmeans” package (Lenth, 2016) of the R software (when statistical difference was significant; De Mendiburu and Yassen, 2020).

Additional univariate and bivariate descriptive statistical tools included calculation of frequencies and percentages, standard deviation, minimum and maximum values and were used to describe the main socioeconomic characteristics as well as the parameters for disease epidemics in the field.

#### Multivariate statistics

A multivariate analysis was performed using a factorial method (Factor Analysis of Mixed Data) to analyze the association between epidemic parameters, detection results and the main seed systems parameters. Hierarchical Clustering on Principal Component (HCPC) method was used to identify groups (or clusters) of fields showing similar characteristics within the study area according to relevant epidemic parameters, detection results and seed systems parameters. For the analysis, the Ward clustering algorithm and the Euclidian distance were used (Hartigan, 1975; Kassambara, 2017).

The “Test Value” criterion (VT) was used to select variables considered as relevant for the characterization of groups/clusters from HCPC analysis. For continuous variables, the VT was used to rank/sort variables according to their relevance in order to distinguish the variables that play an essential role in the interpretation of the groups. For discrete variables, the VT was used to highlight the category which characterizes the better the group/cluster of observations (Lebart et al., 1995; TANAGRA, 2009).

#### Risk factors/determinants of CBSVs

Binary logistic regression (Hosmer and Lemeshow, 2000; Collet, 2003) was used to identify seed system risk factors (determinants) associated with the absence (negative detection) of CBSVs infection in cassava fields.

The probability *p(x)* of a field to be infected by a particular form of CBSVs (*Y* = 1) given a modality *x_1_* of the predictor variable X (1) was estimated by comparing the Odd Ratio (*OR*) of that modality *x_1_* to the reference *x_0_* (2). The possible outcomes of this comparison were interpreted as indicated in the Table 2.


(1) $$p\left(x\right)=P\left(Y=1|X=x\right)$$

(2) $$\left(x_{1},x_{0}\right)=\frac{odds\left(x_{1}\right)}{odds\left(x_{0}\right)}=\frac{\frac{p\left(x_{1}\right)}{1-p\left(x_{1}\right)}}{\frac{p\left(x_{0}\right)}{1-p\left(x_{0}\right)}}$$

**Table 2:** Rules (*Rn*) for interpretation of odd ratios.

*R1*	*OR*(*x*_*1*,_ *x_0_*) > 1→*p*(*x_1_*)*> p* (*x_0_*)
*R2*	*OR*(*x*_*1*,_ *x_0_*) = 1→*p*(*x_1_*) *= p* (*x_0_*)
*R3*	*OR*(*x*_*1*,_ *x_0_*) < 1→*p*(*x_1_*) *< p* (*x_0_*)

Rules for interpretation of OR (*Rn*) are presented in the Table 2. They could be summarized as follow:

R1: When OR > 1, the risk of having an infected field is higher (more probable) in the case X = *x_1_* compared to the reference case X = *x_0_*.

R2: When OR = 1, the risk of having an infected field in the case X = *x_1_* is equal to that of the reference case X = *x_0_*.

R3: When OR < 1, the risk of having an infected field is lower (less probable) in the case X = *x_1_* compared to the reference case X = *x_0_*.

#### Model building process

Upon completion of the bivariate analysis (univariable model), all variables whose univariate test had a value of *p* <0.05 were included in the initial multivariate model along with all variables that were relevant for explaining the studied questions (Mickey and Greenland, 1989; Hosmer and Lemeshow, 2000).

Subsequent models were fitted by sequentially including or excluding variables from that initial model based on statistical criteria using forward and backward stepwise procedures (Chambers, 1992; Venables and Ripley, 2002). Before running the stepwise algorithm, multicollinear variables were visually identified using correlation matrix for predictor variables and ensured they were effectively suppressed from the fitted model after running the stepwise procedure. The likelihood ratio test (LRT or deviance adequation test) was used to assess the fitness of these second-order models compared to the initial one (Hastie and Pregibon, 1992; Hosmer and Lemeshow, 2000). Additionally, all candidate models were assessed for their goodness-of-fit using the Hosmer and Lemeshow test implemented using the “ResourceSelection” package in R and for their stability, sensitivity and specificity using the mplot. The AIC (Akaike Informative Criterion) was used to select the optimal model among the best fitted. All else being equal, the model with the lower AIC was considered as optimal and the corresponding significant predictors were considered as determinant risk factors (Sakamoto et al., 1986).

The VIF (Variance Inflation Factor) was used to detect the presence of multicollinearity within the predictors for the best-fitted model and therefore to assess the suitability of the coefficients for the interpretation (Fox and Weisberg, 2019). Odd Ratios and confidence intervals were computed using Wald method.

Geographical maps were elaborated using Quantum GIS Software version 2.14.0 Essen by interpolating values of disease incidence using the Inverse Distance Weight method (IDW; QGIS Development Team, 2021).

## Results and interpretation

### Identification of clusters within the study area

**Figure 3:**
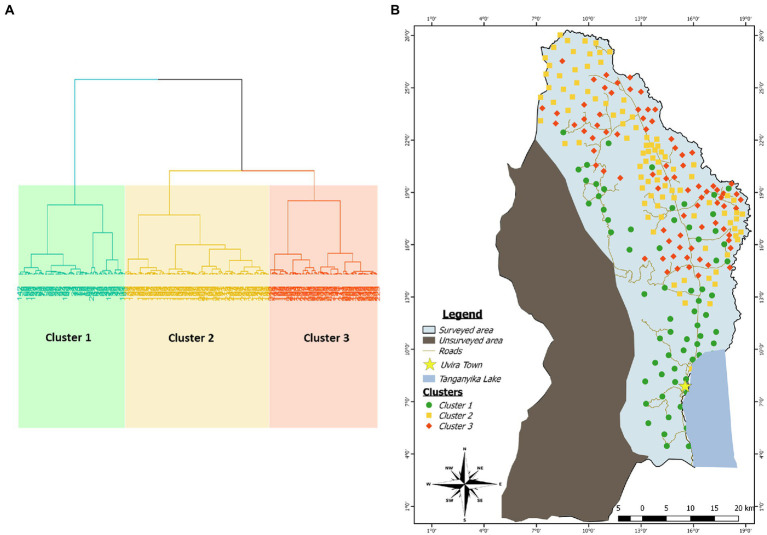
**(A)** Cluster dendogram showing the repartition of the data into three clusters using the Hierarchical Clustering on Principal Component method. **(B)** Mapping of the clusters identified by HCPC in the study area. Each color category is associated to a cluster: Green for cluster 1, yellow for cluster 2 and red for cluster 3.

An HCPC analysis was carried out based on the results of the survey (farmer interview and symptoms observation; see the questionnaires in Supplementary Materials 1–3) and of the molecular detection of both viruses (see next chapter). The nine most important parameters that optimally characterize the studied area were identified with the HCPC analysis. Using those selected parameters (see Table 3 for categorical variables and Table 4 for quantitative variables), the study area was separated into three clusters each with a similar number of fields (Figure 3).

**Table 3:** Categorical variables associated with the description of clusters from HCPC analysis.

Cluster 1 (*n* = 80; 33%)	Cla.Mod^1^	Mod.Cla^2^	Global^3^	Value of *p*^4^	v.test^5^
Cutting pathways = Local fields, Communautary groups	91	39	14	0	7,66
Types of foliar symptoms = No symptoms	69	51	24	0	6,73
Farming system = Monocropping + polycropping	100	15	5	0	4,94
Number of whiteflies = “No whiteflies”	53	38	23	0	3,58
Presence of weeds = No	38	70	60	0,02	2,27
Cluster 2 (*n* = 85; 35%)					
Number of whiteflies = “1–10”	62	86	48	0	8,89
Land tenure = rented	65	69	37	0	7,61
Types of foliar symptoms = Systemic and localized	67	66	34	0	7,55
Cutting pathways = Neighbor countries	100	29	10	0	7,35
Cluster 3 (*n* = 81, 33%)					
Types of foliar symptoms = Systemic and on the whole plant	96	63	22	0	11,16
Land tenure = Owner	50	96	63	0	8,26
Cutting pathways = Local fields	42	86	67	0	4,67
Number of whiteflies = “11–20”	56	33	20	0	3,69
Presence of weeds = yes	43	53	40	0	2,84
Infection status = UCBSV	52	21	13	0,02	2,35
Farming system = Monocropping	37	81	72	0,02	2,26

1 Percentage of individuals showing the characteristic (variable = modality) who belongs to the cluster.

2 Percentage of individuals of from that cluster showing the characteristic (variable = modality).

3 Percentage of individuals showing the characteristic (variable = modality) in the whole population (*n* = 246).

4 Pearson’s Chi squared test; Fisher’s exact test. It assesses the strength of the link between a modality and a cluster. The value of p of a modality is less than 5% when that modality is significantly linked to the cluster that is being interpreted. Only modalities with values of p less than 5% are shown.

5 Test value: transformation of the value of p into a quantile of the normal law. When the V-test is negative, it means that the modality is significantly less present (under-expressed) in that cluster compared to the presence of this modality in the whole dataset (these modalities were not included in the table). However, if the v-test is positive, the corresponding modality is significantly more present (over-expressed) in that cluster (Husson et al., 2011).

**Table 4:** Continuous (quantitative) variables associated with the description of clusters from HCPC analysis.

	In cluster^1^		Overall^2^		Value of *p*	v. test^4^
	Mean	sd^3^	Mean	Sd^3^		
*Cluster 1* (80 fields)						
Plant age [months]	11.2	9.6	3	2.7	2.7e-08	5.6
Mean symptoms incidence (in %)	25	48	14	28	2.1e-15	7.9
*Cluster 2* (85 fields)						
Mean symptoms incidence (in %)	42	48	22	28	0.012	2.5
*Cluster 3* (81 fields)						
Mean symptoms incidence (in %)	74	48	22	28	1.3e-24	10.2
Plant age [months]	8.6	9.7	3	2.7	6.8e-06	4.5

1 Statistics of continuous variables in the cluster.

2 Statistics of continuous variables in the whole subpopulation.

3 Standard deviation.

4 Test Value: transformation of the value of *p* into a quantile of the normal law. When the V-test is negative, it means that the modality is significantly less present (under-expressed) in that cluster compared to the presence of this modality in the whole dataset (these modalities were not included in the table). However, if the v-test is positive, the corresponding modality is significantly more present (over-expressed) in that cluster (Husson et al., 2011).

The cluster 1 is characterized by the presence of a consistent number of fields (91%) that were grown using cuttings that farmers obtained from 2 pathways (i.e., their own fields and the social organizations to which they belong to). In these fields, farmers combined monocropping and crop association farming systems to grow cassava and majority of the plants from this cluster (69%) did not display CBSD symptoms. Fields from cluster 1 were 11-month-old at the time of survey and had CBSD mean symptoms incidence of 25%, significantly lower than the other clusters (Table 4). This cluster also includes high number of fields characterized by the absence of weeds (38%) or whiteflies (53%).

The 10 cassava fields in which farmers used cuttings obtained from neighbor countries are grouped in the cluster 2. These fields are mainly held by the farmers in a tenancy mode (65%). The abundance of whitefly varied between 1 and 10 in most of the fields (62%). Most cassava plants from cluster 2 (67%) displayed CBSD symptoms localized on leaves and/or on the stem.

The cluster 3 is characterized by the presence of CBSD symptoms on both leaves and stems in nearly all fields (96%). Additionally, around 50% of cassava fields are held by farmers in a private ownership. Cassava is grown in monocropping in 37% of the field using cuttings that farmers obtained exclusively from their own fields. The absence of weed was recorded in 57% of the fields while the number of observed whiteflies was higher (11–20) than cluster 2 in 56% of the fields. The average age of cassava plants at the time of survey was 8.5 months. Most of fields from this cluster (52%) were found to be infected by UCBSV and had a mean symptoms incidence higher than what was reported in the two previous clusters (74%).

The spatial arrangement of the above-described clusters is shown on the Figure 3. Fields initially classified as belonging to the site 2 (South) are entirely included in the cluster 1 as it is the case for the majority of fields initially defined as belonging to the site 5 (West). However, there is not a clear spatial demarcation between areas occupied by clusters 2 and 3 despite the fact that much more fields belonging to the cluster 3 presented a tendency to aggregate on the northern part of the border with Burundi. Additionally, the clusters 2 and 3 occupied the areas initially defined as sites 1 (North), 3 (Center) and 4 (East).

### CBSVs detection and symptom incidence within the study area and the clusters

The molecular detection of CBSVs in the region and per cluster is shown in the Table 5. Overall, samples from 77 fields (31.3%) out of the 246 were tested positive for CBSVs (Supplementary Figure 2). The geographical localization is shown in Supplementary Figure 3. Fields infected by CBSV alone were evenly distributed along the survey area while fields infected by UCBSV or by both species were concentrated in the Northern and central parts or in the Eastern part of the surveyed area, respectively. Among infected samples, UCBSV in single infection was the most prevalent (42.9%) followed by single infection with CBSV (35.1%) and mixed infection with CBSV and UCBSV (22,1%).

**Table 5:** Percentages of CBSVs detection and of symptom incidence according to clusters of the study area.

Characteristic	Cluster 1, [80]*^1^*	Cluster 2, [85]	Cluster 3, [81]	Overall, [246]	Value of *p**^2^*
*Infection status* (molecular incidence)					0.021
CBSV	15%	7%	11%	11%	
CBSV+UCBSV	6%	8%	6%	7%	
UCBSV	5%	14%	21%	13%	
Negative	74%	71%	62%	68%	
Mean symptom s incidence (%)	25 c	42 b	74 a	47	
SD	15	22	22	20	
Min	3	7	17	9	
Max	67	83	100	83	

1 [n]: Numbers in brackets represents the number of fields.

2 Pearson’s Chi-squared test.

In addition, the CBSVs’ detection percentage varied significantly across clusters (value of *p* = 0,021). Infection by CBSV was most prevalent within the cluster 1 (15%) while the mixed infection was most present within the cluster 2 (8%). The cluster 3 was characterized by the higher prevalence of UCBSV (21%).

According to the symptoms incidence results (Table 5), the first cluster was characterized by an average incidence (25%) significantly lower than the cluster 2 (42%) which was also significantly lower than cluster 3 (74%). The number of fields for each level of symptoms severity score across the three clusters as well as the number of fields according to symptom types is shown in the Table 6. Results suggested that most fields belonging to the cluster 1 had typical CBSD symptoms located on lower leaves (41 plants out of 80). This was not the case for the two other clusters where symptoms were rather absent in most fields (cluster 2: 56 fields out of 85) or scattered on the whole plant (systemic-like symptoms, on both leaves and stems) in the cluster 3. Results further suggests that differences in proportions of fields for each modality of foliar symptoms across clusters are statistically significant (value of *p* Pearson’s Chi-squared test = 0,001). Symptom severity score of 1, 2 and 3 included most fields in the survey (82%) with significant differences between clusters. For example, 82 % of the fields presented a severity score of 1 or 2 for the cluster 1 while they represented 63 and 27% of the fields for cluster 2 and 3, respectively. Cluster 3 was characterized by a higher proportion of field with severe symptoms (4 and 5 scores): 33% compared to 10 and 9% for cluster 1 and 2, respectively.

**Table 6:** Types and severity of foliar symptoms observed on surveyed plants across clusters.

Characteristic	Clusters			Overall (*N* = 246*^1^*)	Value of *p**^3^*
	Cluster 1 [*N* = 80]^1^	Cluster 2 [*N* = 85]	Cluster 3 [*N* = 81]		
Foliar symptoms types^2^					<0.001
LL	51%	14%	7%	24%	
NO	31%	66%	4%	34%	
SL	15%	20%	26%	20%	
SW	3%	n.a.^4^	63%	22%	
Severity score^5^					<0.001
1	37%	26%	2%	20%	
2	45%	39%	25%	35%	
3	9%	26%	40%	26%	
4	4%	0%	22%	0.1%	
5	5%	9%	11%	0.1%	

1 [n]: Numbers in brackets or parentheses represents the number of fields.

2 Types of foliar CBSD symptoms based on distribution of leaf chlorosis and stem lesions on the plant: systemic and on the whole plant (SW), systemic on leaf or stem parts but localized (SL), only on lower leaves (LL).

3 Pearson’s Chi-squared test.

4 Not applicable. It means that the modality related to this infection type was not observed.

5 Foliar symptom severity score based on 1–5 scale (Alicai et al., 2016): 1 = No visible symptoms (not shown in Table 4), 2 = mild vein yellowing or chlorotic blotches on some leaves, 3 = pronounced/extensive vein yellowing or chlorotic blotches on leaves but no lesions or streaks on stems, 4 = pronounced/extensive vein yellowing or chlorotic blotches on leaves and mild lesions or streaks on stems, 5 = pronounced/extensive vein yellowing or chlorotic blotches on leaves and severe lesions or streaks on stems, defoliation and dieback.

### Pathways of cuttings used by farmers

Cuttings used for planting cassava fields were obtained from diverse pathways (Table 7). Pathways that required no or less charges and located in the closest farmer’s environment (representing no or low charges, without travelling long distances and involving actors closely related to the farmer) were the most used. These corresponded to cuttings provided by the farmer himself (obtained from own fields grown with cassava or fields from the neighbors) as well as cuttings obtained from cooperatives to which the farmer belongs or could access (associations, cooperatives, Non-governmental Organization-NGO-, peasant local associations etc.). Unless the fact that some farmers used cuttings from only one pathway (cuttings originated rather exclusively from farmer’s own production: 22% or exclusively from cooperatives: 3%), most of farmers used a combination of different pathways to access cuttings (75%). In fact, the situation in the surveyed area suggested that most of farmers used a combination of 2 or 3 pathways to obtain cuttings.

**Table 7:** Proportion of fields grown by types of cassava varieties from different pathways.

Characteristic	Local varieties [1]*^1^*	Improved varieties [126]^3^	Both [119]	Overall [246]*^1^*	Value of *p**^2^*
*Cutting pathways*					0.4
Farmers (F)	-	23% _[27]_	23% _[27]_	23% _[54]_	
F + Cooperatives (C)	100% _[1]_	44% _[53]_	41% _[49]_	43% _[103]_	
F + C + Market	-	15% _[18]_	8% _[10]_	12% _[28]_	
F + C + Multiplier	-	17% _[20]_	26% _[31]_	21% _[51]_	
F + Neighbor countries	-	1.7% _[2]_	2% _[2]_	2% _[4]_	

1 [n]: Numbers in brackets represents the number of fields.

2 Pearson’s Chi-squared test.

-The modality related to this cutting pathway was absent.

3 No data on the pathways used to obtain cuttings of improved varieties grown in 6 fields could be obtained.

The table presented in Supplementary Table 1 summarizes the proportion of fields grown with cuttings obtained from each category of pathways identified as well as the means used by farmers to obtain cuttings across clusters. Globally, 103 surveyed fields were grown with cuttings that farmers obtained from a dual pathway source: from their own fields (and fields from neighboring farmers) and from social organizations they belong to. Fifty-four fields were also identified as being grown exclusively with cuttings that farmers obtained from their own fields (or fields of neighboring farmers). Cuttings originating from seed multipliers were found to be grown in 51 fields, always in combination with cuttings originating from farmer’s own fields and Cooperatives. Majority of fields grown with cuttings from seed multipliers were in cluster 2 (39 fields). Few fields (4) were found to be grown with cutting originating from neighbor countries.

Cassava fields grown by cuttings that farmers obtained exclusively from their previously grown fields were mostly located in the cluster 1 (26 fields out of 54) while cuttings resourced from Cooperatives pathways are mostly found in the cluster 3 (44 fields out of 103). Cutting obtained from Market (18 fields out of 28) and from Seed multiplier (20 fields out of 51) were mostly grown in the cluster 2. Results further suggested that differences observed in proportions of fields grown by cuttings from different pathways across sites were not different (Pearson’s Chi-squared test = 0.04).

Results further suggested that all farmers (100%) have used cuttings obtained for free while nearly half of them (48%) have paid for cuttings. Other sources of cuttings were obtained by working in the field of other farmers (6%) or by sharing production after harvest (10%; Supplementary Table 1).

The Table 7 also shows the proportion of fields grown by different types of cassava varieties from each of the pathways described above. Generic names of improved varieties are most of the times changed by farmers during the adoption process to adapt them to local dialects. During the survey, the challenge was to identify a variety as local or improved despite the local name assigned by farmers. Physical traits or appearance of cassava plants were mainly used to determine if a variety was local or an improved one. This identification strategy was rendered efficient by including into the survey team local agronomist officers able to identify varieties in the field. Local names of all the varieties (improved as well as local) identified during this work are shown in the table presented in Supplementary Table 2. Results suggested that most of fields were grown either with improved varieties only (51%), either with a mixture of improved and local varieties (48%; Table 7). A single field was grown exclusively by local varieties.

The table presented in Supplementary Table 3 summarizes the proportion of each type of infection according to cutting pathways. Results suggested that among fields that tested positive to UCBSV infection (34 fields), more than the half (19 fields) were grown by cuttings originated from the dual source Cooperatives + farmers. Among fields that tested positive to CBSV infection (28 fields) and to mixed CBSV+UCBSV infection (18 fields), majority of them were grown with cutting originating from a dual (farmer’s + Cooperatives) and exclusively from farmer’s pathways (respectively 17 fields out of 28 for CBCSV infection, and 13 fields out of 18 for mixed CBSV+UCBSV infection). On the other side, the overall proportion of fields free from infections is higher (78%) when they are grown using cuttings issued from seed multipliers.

### Seed system risk factors associated with CBSD

After fitting an initial model containing 37 candidate predictors (most of which were studied in previous sections), a final model containing 9 predictors, all statistically significant, was optimized using a combined forward + backward stepwise procedure (Chambers, 1992; Venables and Ripley, 2002). The results obtained are summarized in the Table 8.

**Table 8:** Prediction of risk factors associated with CBSD (based on RT-PCR detection).

Characteristic		Bivariate statistics			Prediction		
	Absence of infection [98]*^1^*	Presence of infection [50]	Overall [148]	Value of *p**^2^*	OR*^3^*	95% CI*^3^*	Value of *p*
Assistance/support by extension services				n.s.^4^			0.05
No	62% [43]	38% [26]	100% [69]		1.00	*Reference*	
Yes	70% [55]	30% [24]	100% [79]		0.32	0.08, 1.03	0.041
Knowledge of cassava pests and diseases				n.s.^4^			0.002
No	43% [3]	57% [4]	100% [7]		1.00	*Reference*	
Yes	67% [95]	33% [46]	100% [141]		29.1	3.23, 355	0.004
Knowledge of management practices				0.064			0.008
Yes	77% [34]	23% [10]	100% [44]		1.00	*Reference*	
No	62% [64]	39% [40]	100% [104]		0.14	0.02, 0.62	0.016
Which distance to acquire cuttings?				0.5			0.001
Very close (<1 km)	71% [49]	29% [20]	100% [69]		1.00	*Reference*	
Close (1–5 km)	60% [12]	40% [8]	100% [20]		0.96	0.23, 4.22	n.s.^4^
Far (5–10 km)	59% [16]	41% [11]	100% [27]		0.3	0.66, 2	n.s.^4^
Very far (>10 km)	66% [21]	34% [11]	100% [32]		0.08	0.02, 0.33	0.001
Proximity to town (Uvira)				0.5			0.036
Very Close (<1 km)	75% [6]	25% [2]	100% [8]		1.00	*Reference*	
Close (1–5 km)	78% [18]	22% [5]	100% [23]		0.59	0.03, 7.51	n.s.^4^
Far (5–10 km)	66% [23]	34% [12]	100% [35]		0.12	0.01, 1.26	n.s.^4^
Very Far (>10 km)	62% [51]	38% [31]	100% [82]		0.09	0.00, 0.85	0.061
Proximity to borders				n.s.^4^			0.05
Very Close (<1 km)	68% [39]	32% [18]	100% [57]		1.00	*Reference*	
Close (1–5 km)	67% [28]	33% [14]	100% [42]		1.16	0.56, 2.41	n.s.^4^
Far (5–10 km)	65% [20]	36% [11]	100% [31]		2.07	0.82, 5.31	n.s.^4^
Very Far (>10 km)	61% [11]	39% [7]	100% [18]		4.45	1.30, 17.4	0.023
Methods used to manage CBSD				0.027			0.001
Use cuttings from symptomless plants	76% [34]	25% [11]	100% [45]		0.43	*Reference*	
Use local varieties	85% [29]	15% [5]	100% [34]		1.00	0.97, 5.86	n.s.^4^
Use certified varieties	53% [23]	45% [20]	100% [43]		2.25	0.89, 5.89	0.001
Cutting pathways				n.s.^4^			0.001
Farmers (F)	57% [20]	43% [15]	100% [35]		1.00	*Reference*	
F + Cooperatives (C)	64% [41]	36% [23]	100% [64]		2.06	0.55, 7.81	n.s.^4^
F + C + Market	67% [4]	33% [2]	100% [6]		10.7	0.56, 272	n.s.^4^
F + C + Multiplier	75% [30]	25% [10]	100% [40]		7.96	1.55, 53.1	0.019
F + Neighbor Country	100% [4]	0% [0]	100% [4]		6.051	0.00, NA	n.s.^4^
(Intercept)					17	0.49, 700	0.12

1 [n]: numbers in brackets represents the number of fields.

2 Pearson’s Chi-squared test; Fisher’s exact test.

3 OR = Odds Ratio, CI = Confidence Interval.

4 n.s. = the value of *p* is >0.05.

Results showed that cassava fields owned by farmers who received supports (training, advising or field visit) and had a certain knowledge of cassava pests and diseases as well as on management practices against CBSD, are significantly more likely to be free from CBSVs infection compared to fields belonging to the other farmers. Beyond these aspects related to farmer’s awareness, factors related to the distance location of cassava fields were also found to significantly impact the outcome of CBSD infections. In fact, cassava fields located very far (more than 10 Km) from the borders as well as from Uvira town were significantly and highly associated with the absence of CBSD compared to fields that were very close (less than 1 km) to borders and Uvira (Value of *p* < 0.05). Also, fields grown with cuttings obtained by farmers from very far locations (more than 10 km) appeared to be significantly less prone to CBSVs infections compared to fields grown with cuttings that farmers obtained in nearby locations (less than 1 Km; value of *p* = 0.05). Additionally, fields in which farmers were using certified varieties to grow cassava were significantly and highly associated to the absence of CBSD compared to fields grown with cuttings taken from asymptotic plants (value of *p* < 0.005). Results further suggest that when farmers envisage the option of using cuttings from seed multipliers pathways to grow cassava, the risk of having infection with CBSVs in their fields is significantly lower (less probable; value of *p* < 0.05) compared to situations where fields where grown with cuttings taken exclusively from farmer’s own fields.

## Discussion

The preliminary description of the study area based on socioeconomic, ecological and agronomic parameters supported the existence of various sites (Table 1; Figure 2). In addition, to highlight the role of seed cutting pathways in the epidemiology of CBSD, this information was completed by plant disease observation and virus detection. Such phytopathological information opened the possibility of associating CBSD-infected plants obtained from a particular seed pathway to a set of explanatory parameters depicting the environmental context that, we hypothesized, could explain the outcome of infection. Even tough symptoms presence on a plant is a proxy of infection, they can be misleading in certain circumstances particularly when they are not specific as it is the case for the CBSD. It appeared therefore rationale to use molecular diagnosis so that errors due to the misidentification of the causal agent could be significantly lowered. This integration of several multidisciplinary data facilitated an in-depth description of the study area regarding the objectives of the study and allowed designing local-adapted approaches to act at the formal-informal interface of the cassava seed system for the mitigation of the CBSD dissemination.

Before discussing the results, it is important to state that, despite the multidisciplinary approach used in this study, our survey, as any survey, presented some limitations that might introduce bias on the conclusion drawn. For example, the use of some pre-selected questions in the questionnaire, the random sampling and observation on field (impacting sample representativeness) as well as the limited number of observations. In addition, we were not allowed to uproot cassava plants, and therefore to observe necrotic symptoms on root, in 86% of the surveyed field.

The molecular diagnostic revealed the presence of the two viral species known to cause the disease in single or mixed infection. The prevalence of both species was similar, 18% for CBSV and 20% for UCBSV. Nevertheless, the analysis of prevalence for each cluster revealed contrasted situations: CBSV prevalence was between 17% (cluster 3) and 21% (cluster 1) while UCBSV prevalence was more heterogeneous ranging from 11% (cluster 1) to 20% (cluster 3).

Epidemiologically, the identification of three clusters made sense. Indeed, the homogeneous cluster 1 gathered most of villages located in the high-altitude zone where the fields presented the lowest incidence of symptom and virus detection (with CBSV the most prevalent virus) while the whiteflies were very rare. On the other side, a heterogeneous zone in the low altitude area (cluster 2 and 3) was characterized by the higher prevalence of UCBSV-infected fields but with distinct symptoms incidence and cutting pathways. In the cluster 2, most diseased fields showed systemic symptoms that were localized either on leaves or stems and that presented a higher incidence than in the cluster 1. In addition, among fields grown with cuttings originating from seed multipliers pathways, a consistent number of them were found in the cluster 2. In the cluster 3, most cassava fields were grown in monoculture, most of them were colonized by weeds and presented the highest number of whiteflies, virus infection as well as the highest symptoms incidence rate while presenting typical systemic-like symptoms on the plant. Previous studies have shown that growing conditions (temperature, rainfall and altitude) can induce variation in the expression of foliar symptoms of CBSD (Mohammed et al., 2012) as well as in the dynamic of whiteflies (E. Bisimwa et al., 2012) thus globally reducing disease incidence as observed for the cluster 1. The presence of CBSD-like symptoms on lower leaves is often misleading because symptoms can be confused with those due to normal leaf senescence, and the prevalence of symptoms type restricted on lower leaves could not be necessarily considered as indicative of virus infection. The high prevalence of infected fields in the low altitude areas (clusters 2 and 3) as well as their high symptoms incidence and severity is in agreement with previous findings suggesting that the disease pressure was decreasing with the rise of altitude (Bigirimana et al., 2011; Patil et al., 2011; Bisimwa et al., 2012; Mohammed et al., 2012; Legg et al., 2015; Muhindo et al., 2020).

Furthermore, it is important to note that, in the clusters 2 and 3, the symptom incidence was higher than the virus incidence (percentage of fields tested positive to RT-PCR). This situation suggested that there were a consistent number of plants with symptoms but tested negative by RT-PCR. This situation could be explained by the non-specificity of CBSD symptoms that are reported to be an inconsistent way of identification of CBSVs (Rwegasira and Rey, 2012b). The link between field symptoms and molecular diagnostic could have been better investigated if symptoms assessment were conducted on below-ground parts of the plant as it could provide more reliable data than those collected following leaves and stem observation (Ogwok et al., 2015). Indeed, diseased cassava plants can sometimes recover and become asymptomatic for the above-ground organs whereas roots continue to degenerate. Despite the fact that we initially intended to capture this important information, a consistent number of farmers could not grant authorization for uprooting plant inside their fields. As a consequence, root symptoms were collected only from 33 fields out of the 240. We did not integrate this information in the study as the sampling was not representative. Additionally, the primers used for virus testing have been designed to amplify all isolates whose genome or gene sequence is known. While knowing that CBSV and UCBSV genomes are reported to evolve rapidly under high disease pressure (Ndunguru et al., 2015; Alicai et al., 2016) it is possible that CBSVs isolates not detected by our protocol are present in these clusters. These findings underpins an urgent need to unveil full genomes of CBSVs isolates in this area to support designing inclusive primers necessary for an efficient diagnostic (Adams et al., 2018). High throughput sequencing technology (Massart et al., 2014) can contribute to decipher this problem and will help designing new primers required for increasing the inclusivity of molecular tests. In addition, the viral species complex causing symptoms on cassava might not be fully understood so far. For example, two new viral species belonging to the genus Ampelovirus (MEaV1 and MEaV2) were previously described for the first time infecting cassava plants and one of these species was detected in the area where this study was conducted (Uvira territory; Kwibuka et al., 2021). Nevertheless, the association of these viruses with disease symptom is not yet studied, so the phytosanitary risk posed by them is not known yet.

As suggested in this study, cutting pathways would be determinant in the outcome of CBSVs infections through the fact that when farmers took the option of using cuttings provided from seed multipliers, additionally to cuttings taken from other pathways, the prevalence of CBSVs infection lowered significantly. However, additive effects, resulting from the fact that several pathways were used at the same time by most farmers, might have been obscuring the precise role of each cutting pathway. The decision of using a particular cutting pathway to access cassava planting material by a farmer can result from various considerations that were not investigated in this study. We therefore could not explain why for instance in the cluster 1 a consistent proportion of fields were grown with cuttings originating from previously grown fields while in cluster 2 the situation was quite different (a consistent number of fields were grown with cuttings originating from cooperatives). However, we could observe that, for example, a consistent number of fields in the cluster 1 were located a bit far from main roads/rails than fields from cluster 2. Such distant location pattern of cassava fields could narrow the possibilities in the sourcing of cuttings since the transportation of vegetative material over long distances can be a serious limit for the farmer as seen in the literature (El-sharkawy, 2004; Mdenye et al., 2016; Kidasi et al., 2021).

Improved varieties are widely used. This is consecutive to previous interventions for mitigating devastations caused by the CMD epidemics in this area. Local varieties were progressively abandoned before the outbreak of CBSD against which most of improved CMD-resistant varieties are susceptible. Currently, the research for developing CBSD-tolerant varieties is ongoing (Sheat et al., 2019; Manze et al., 2021). Nevertheless, due to a scarcity of healthy cuttings and disappointments often encountered after using cuttings expected to be healthy, farmers return to traditional varieties that they have been using for years. This scarcity of healthy cuttings is therefore primarily due to the lack of multiplication of phytosanitized tolerant varieties that are available as well as to the lack of varieties with dual resistance to CBSD and CMD for multiplication and dissemination. The use of local agronomists was privileged in this study in the frame of a simple differentiation between local and improved varieties grown on field. The use of molecular markers to perform morphological identification, using for example SNP markers (Karim et al., 2020; Pierre et al., 2022), would have brought more precise information for confirming identities of cassava genotypes. This strategy would provide further evidence on the possible linkages between CBSD symptomology and cassava genotypes but we did not definitely envisage it in this study considering the resources and time available. We therefore strongly recommend to future studies to complement viral survey by the genotyping of the harvested plants.

Factors related to farmer’s awareness, especially the assistance from extension services, the knowledge of cassava pests and disease, the knowledge of management practices of CBSD as well as the use of certified varieties, are key determinants to limit CBSVs infection as illustrated by studies conducted elsewhere (Kumakech, 2013; Chipeta et al., 2016; Legg et al., 2017). This is absolutely relevant because being aware of CBSD, its symptoms as well as management practices helps farmers in identifying diseased plants and taking appropriate decisions for managing and mitigating the disease. This is supported by the fact that using cuttings from seed multipliers lowered significantly the risk of CBSD. Here, it is very important to emphasize that seed multipliers benefited more from extension services supports and seemed to develop more skills and knowledge than ordinary farmers. Therefore, intensifying actions aiming at raising farmers awareness on CBSD control would constitute an effective option to mitigate the disease.

It was also very interesting to found that parameters related to the geographical location of fields, particularly in relation with the national border, were significantly associated with the outcome of CBSD infection. At this stage we do not know if this association is causative or spurious, border proximity hiding other factors. In addition, only 4 farmers mentioned neighboring countries as pathways of seeds. Nevertheless, it would be very important to mention that the proportion of fields effectively grown with cuttings originating from neighbor countries might be higher than what was reported in this study because the unauthorized transboundary movement of planting material is forbidden, therefore despite guarantees of non-divulgation of information, respondent might not explicitly admit to be engaged in such kind of exchanges. Such phenomenon of underestimation of transboundary movement of planting material has been already suggested (Kumar et al., 2021). Therefore, if this association is further demonstrated as causative, farmers living along borders between D.R. Congo, Burundi and Rwanda should be particularly targeted for awareness raising on importance of avoiding moving cassava planting material across borders without following proper international regulations (IPPC, 2021).

Mapping epidemiological aspects of a disease in a range of environments can identify locations where investments in extension and farmer support are most likely to be effective (Buddenhagen et al., 2017). Results provided in this study suggested that efforts to promote a clean seed system in the study region could therefore target the areas covered by the cluster 1 and use it as multiplication site due to its low disease pressure and low vector population density. Additionally, in the same analysis, efforts of extension work should be focused on raising farmer awareness of CBSD to sustain the effectiveness of control strategies. Such extension efforts must target both local and national organizations involved in the farmer’s Cooperatives and would put much attention on areas covered by clusters 2 and 3.

The use of improved varieties in the previous years did not guarantee effective protection against CBSD as observed in the study area because most of these varieties were tolerant to CMD only; viruses kept multiplying inside their vegetative tissues thus leading to increased virus load over years (Manze et al., 2021). Therefore, there is an urgent need to insist on the application of a rigorous phytosanitation program to ensure planting material will be subjected to a cleaning process that will lower the virus load. This will necessitate a good expertise in plant virus diagnostic to ensure that sensitive and inclusive tests are applied on elite materials before they could be multiplied and supplied to farmers. These interventions must also include local (traditional) varieties and could contribute to reduce the disease pressure observed into clusters 2 and 3. A key element on which an adapted program of integrated pest and disease management (IPDM) is based for reducing the disease severity and preventing a disease from further spread include the use of healthy/resistant varieties. Within the surveyed country, existing organizations involved/in charge of developing and supplying healthy seeds and regulating activities within the formal seed sectors include local, national as well as international organizations/institutions. However, the predominance of informal actors within this seed system (private seed sellers, village seed multipliers etc.) has the advantage of disseminating seeds where some bigger institutions and even public sector does not reach out as previously illustrated (Beyene, 2010; Pandit et al., 2011). This capacity of delivering seeds on the last mile of the territory is limited by the lack of access to “virus-free” plants. Information about the collaborations between formal and informal actors as well as their respective interventions in the seed system could therefore allow to identify limitations encountered by all of these actors. However, such information was not captured by this study and future works could consider integrating these observations.

Results from this study further suggested that it would be important to empower and to promote local cassava-seed multipliers, particularly in the cluster 1, as they have been shown to be more reliable in delivering disease-free materials. However, it was shown that farmers had to travel more than 10 kilometers to access less risky cuttings. This could mean that the number of actors susceptible of delivering good-quality planting material to farmer’s is still too low. This could represent an opportunity to draw farmer’s attention to business opportunities offered through cassava seed system activities, thus giving a scope to turn this activity commercially attractive in this area.

In this context, promoting the cassava seed system will undoubtedly raise the need of reinforcing the mechanisms of controlling seed multiplication fields. This could be done by rigorous inspections by well-trained inspectors as well as by testing elite cassava materials used for propagation using sensitive molecular techniques preferably on-site (Tomlinson et al., 2013). However, this will raise the question of sustainability due to the high costs involved in running such techniques. Fortunately, sensitive as well as easy-to-use kits [such as Lateral Flow devices (LFDs) or RT-LAMP] that can be implemented on field and that require reasonable consumables, resources and instruments have been developed (Tomlinson et al., 2013) and, after proper training and validation, could allow local seed multipliers to directly identify healthy mother plants candidate for multiplication and dissemination. This could be an additional aspect where expertise from local Universities would be crucial in supporting the establishment of an efficient cassava seed system.

## Data availability statement

The original contributions presented in the study are included in the article/Supplementary Material; further inquiries can be directed to the corresponding authors.

## Author contributions

SM, HV, EB, and YK: conceptualization. SM, YB, and YK: methodology. SM, HV, EB, YB, and LL: validation. YB and YK: formal analysis. YK: investigation and writing—original draft preparation. SM, HV, EB, and LL: resources and supervision. SM and HV: funding. All authors contributed to writing—review and editing, contributed to the article, and approved the submitted version.

## Funding

The work of YK, CN, and JB in D.R. Congo was supported by the iCARE (Improved Cassava Virus Resistance mitigation strategies and development of disease-free seed system) project funded by ARES (Académie de Recherche et d’Enseignement Supérieur, Fédération Wallonie-Bruxelles, Belgium) and granted to HV.

## Conflict of interest

The authors declare that the research was conducted in the absence of any commercial or financial relationships that could be construed as a potential conflict of interest.

## Publisher’s note

All claims expressed in this article are solely those of the authors and do not necessarily represent those of their affiliated organizations, or those of the publisher, the editors and the reviewers. Any product that may be evaluated in this article, or claim that may be made by its manufacturer, is not guaranteed or endorsed by the publisher.

## Supplementary material

SUPPLEMENTARY MATERIAL 1 Questionnaire used for the epidemiological survey in cassava farmer’s fields.

SUPPLEMENTARY MATERIAL 2 Questionnaire used for farmers interviews on seed systems.

SUPPLEMENTARY MATERIAL 3 Questionnaire used for interview of farmers specialized in cassava seed multiplication.

SUPPLEMENTARY FIGURE 1 Schematic representation of a cassava field showing the selection strategy and process for symptomatic plants observation and sample collection.

SUPPLEMENTARY FIGURE 2 Results from molecular detection showing the agarose gel with amplicons: Detection of CBSV (~703 bp) and UCBSV (~800 bp) in samples from the site 1 (1 and 6), site 2 (2 and 7), site 3 (3 and 8), site 4 (4, 5 and 9) and site 5 (10, 11 and 12). 1 kb molecular ladder (L) is shown on the left side of the gel. Positive (C+) and negative controls (C-). Numbers 1 to 12 are representative samples retained for the illustration.

SUPPLEMENTARY FIGURE 3 Geographical mapping of results from molecular detection.

SUPPLEMENTARY TABLE 1 Proportion of fields grown by different types of cutting pathways and means employed by farmers to obtain cuttings across clusters.

SUPPLEMENTARY TABLE 2 Number of fields grown by improved as well as local cassava varieties identified in each cluster.

SUPPLEMENTARY TABLE 3 Proportion of healthy and infected fields for each type of infection according to the channels used to obtain cutting.
