# Supplementary material for: Risk factors associated with cassava brown streak disease dissemination through seed pathways in Eastern D.R. Congo
Source: Front Plant Sci. 2022 Jul 22;13:803980. doi: 10.3389/fpls.2022.803980 (PMC9354974; doi:10.3389/fpls.2022.803980)
Supplement: SUPPLEMENTARY MATERIAL 1 — Questionnaire used for the epidemiological survey in cassava farmer’s fields. [file Data_Sheet_1.zip › Supplementary material/Supplementary Material 1.pdf]

supplementary material 1

Epidemiology 2019 OK

1. Nom de l'enquêteur

____

2. Numéro du champ

Ecrire le numéro du champ en continuant à partir du numéro du dernier champ de la journée précédente

____

I. LOCALISATION DU CHAMP

I.1. Territoire

____

I.2. Nom du village/quartier

____

I.3. Coordonnées géographiques du champ

Se mettre au milieu du champ

latitude (x.y °)

____

longitude (x.y °)

____

altitude (m)

____

précision (m)

____

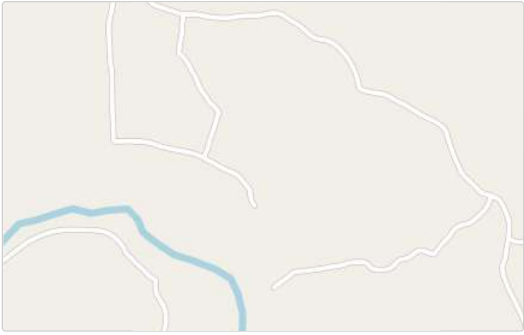

II. Caractéristiques foncières

II.1. Quelle est la superficie de votre champ en hectares

____

II.2. Quel est l'âge des plants de manioc dans le champ? en mois

____

III. SYSTEME DE CULTURE

III.1. Système de culture du manioc

- [ ] Monoculture
- [ ] Association

III.2. Si Association culturale, quelles sont les autres cultures associées au manioc?

- [ ] Arachide
- [ ] Haricot
- [ ] Maïs
- [ ] Patate Douce
- [ ] Riz
- [ ] Sorgho
- [ ] Bananier
- [ ] Oignons
- [ ] Tomates
- [ ] Autres cultures

Si "Autres cultures", citez-les

____

III.3. Y a-t-il d'autres espèces de plantes aux alentours du champ de manioc?

- ◯ Oui
- ◯ Non

III.4. Si oui, quelles sont ces espèces?

- ☐ Arbres
- ☐ Manioc
- ☐ Maïs
- ☐ Sorgho
- ☐ Riz
- ☐ Haricot
- ☐ Bananier
- ☐ Patates douces
- ☐ Arachides
- ☐ Oignons
- ☐ Tomates
- ☐ Autres plantes

Si "Autres plantes", citez celles-ci

III.5. Y-a-t-il des espèces de mauvaises herbes dans le champ de manioc?

- ◯ Oui
- ◯ Non

III.6. Si oui, de quel(s) espèce(s) s'agit-il? (le nom vernaculaire ou scientifique).
Se limiter au nom de famille si c'est compliqué

IV. ÉCHANTILLONNAGE MANIOC

1

* **IV.1. Numéro de l'échantillon**
Commencer par le numéro du champ puis mettre / puis le numéro correspondant à l'échantillon de champ

---

IV.2. Nom de la variété
Nom de variété de l'échantillon à collecter

---

* **IV.3. Sévérité des symptômes CBSV sur les feuilles**

- ◯ Pas de symptômes visibles
- ◯ Légères mosaïques foliaires sur certaines feuilles + pas de lésions sur tiges
- ◯ Mosaïques foliaires + légères lésions sur tiges + pas de dieback
- ◯ Mosaïques foliaires + lésions prononcées sur tiges + pas de dieback
- ◯ Défoliation + lésions prononcées sur tiges + Dieback

* **IV.4. Image des symptômes très sévères sur les feuilles et tiges**

Cliquez ici pour téléverser un fichier. (< 5MB)

* **IV.5.Type des symptômes foliaires de la CBSV**
Basé sur la distribution de la chlorose foliaire et des lésions de la tige sur la plante

- ◯ Systémique et sur toute la plante
- ◯ Systémique mais localisée sur les feuilles ou les tiges
- ◯ Seulement sur les feuilles inférieures
- ◯ Pas de symptômes foliaires

*** IV.6. Sévérité des symptômes MAM sur les feuilles**

- ◯ Pas de symptômes visibles
- ◯ Distortion légère seulement à la base des feuilles apparaissant vertes et saines/Légère aspect chlorotique sur l'ensemble des feuilles
- ◯ Aspect de mosaique conspicieux lelong de la feuille + Rétrécissement et distortion du tiers des feuilles
- ◯ Mosaique sévère + distortion de 2/3 des feuilles + réduction générale de la taille des feuilles
- ◯ Mosaique sévère + distortion de 3/4 des feuilles

*** IV.7. Image des symptômes très sévères MAM feuilles**

Cliquez ici pour téléverser un fichier. (< 5MB)

IV.8. Sévérité des symptômes CBSV sur les tubercules

- ◯ Pas de nécrose
- ◯ Lésions nécrotiques légères (1-10%)
- ◯ Lésions nécrotiques prononcées (11-25%)
- ◯ Lésions nécrotiques sévères (26-50%)
- ◯ Lésions nécrotiques très sévères (>50%)

*** IV.9. Images Symptômes très sévères tubercules**

Cliquez ici pour téléverser un fichier. (< 5MB)

*** IV.10. Nombre des mouches blanches sur les feuilles**

- ◯ Absent
- ◯ 1-10
- ◯ 11-20
- ◯ 21-30
- ◯ 31-40
- ◯ 41-50
- ◯ plus de 50

*** IV.11. Numéro de l'identifiant de l'échantillon de manioc**

Il s'agit du numéro de l'autocollant qui sera collé sur l'échantillon prélevé

____

V. Incidence CBSV dans le champ

Nombre des plantes ayant des symptômes similaires à ceux de CBSV sur 30 plants considérées sur le diagonal

____

VI. Incidence CMD dans le champ

Nombre des plantes ayant des symptômes similaires à ceux de la MAM sur 30 plants considérées sur le diagonal

____

VII. ÉCHANTILLONNAGE MAUVAISES HERBES ET ESPÈCES AVOISINANTES

VII.1. Noms des mauvaises herbes échantillonnées dans le champ de manioc

Si plus de deux espèces, séparer les noms par des virgules. les échantillons des mauvaises herbes sont placés dans l'herbier en prenant soin de mentionner le numéro du champ d'origines

____

VII.2. Noms des espèces des plantes échantillonnées dans le voisinage du champ de manioc

Si plus de deux espèces, séparer les noms par des virgules. les échantillons des mauvaises herbes sont placés dans l'herbier en prenant soin de mentionner le numéro du champ de référence

____
