# Supplementary material for: Risk factors associated with cassava brown streak disease dissemination through seed pathways in Eastern D.R. Congo
Source: Front Plant Sci. 2022 Jul 22;13:803980. doi: 10.3389/fpls.2022.803980 (PMC9354974; doi:10.3389/fpls.2022.803980)
Supplement: SUPPLEMENTARY MATERIAL 1 — Questionnaire used for the epidemiological survey in cassava farmer’s fields. [file Data_Sheet_1.zip › Supplementary material/Supplementary Material 2.pdf]

Supplementary Material 2 **SEED SYSTEM: Farmers ok**

0. Numéro du questionnaire (Commencer par SSF):

____

Nom de l'enquêteur

____

I. LOCALISATION DU LIEU DE L'INTERVIEW

I.1. Territoire

- ◯ Uvira
- ◯ Walungu

I.2. Chefferie

- ◯ Bavira
- ◯ Barundi/Plaine de la Ruzizi
- ◯ Bafuliiru

1.3. Nom du village

Le nom du village où l'interview se déroule

____

I.5. Coordonnées Géographiques du lieu d'interview

latitude (x.y °)

____

longitude (x.y °)

____

altitude (m)

____

précision (m)

____

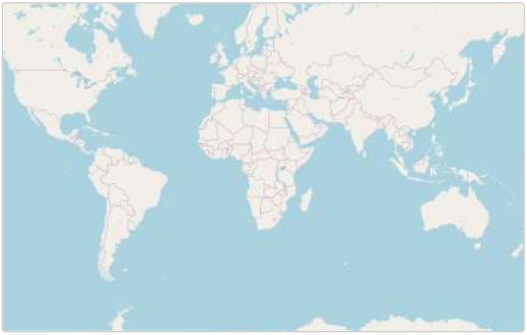

II. IDENTITÉ DE L'AGRICULTEUR

II.1. Nom de l'Agriculteur

____

II.2. Sexe

- ◯ Homme
- ◯ Femme

Age de l'agriculteur

____

II.3. Etat Civil de l'agriculteur

- ◯ Marié
- ◯ Célibataire
- ◯ Divorcé
- ◯ Veuve
- ◯ Séparé

II.5. Quel est votre niveau d'éducation?

- ◯ Primaire
- ◯ Sécondaire
- ◯ Université
- ◯ Professionnel/Metiers
- ◯ Pas étudié

III. CARACTÉRISTIQUES FONCIÈRES

III.1. Quelle est la superficie totale des champs cultivés sous manioc [ha]

____

III.2. Propriété du champ

L'agriculteur est-il propriétaire des champs?

- ◯ Propriétaire
- ◯ Locataire
- ◯ Propriétaire pour certains champs et Locataire pour d'autres

IV. SOCIO ECONOMIQUE

IV.1. En quoi est-ce que la culture de manioc améliore ta vie et celle de ton ménage?

Comment est-ce que la culture de manioc améliore ta vie?

- ☐ Frais scolaires
- ☐ Soins de santé
- ☐ Alimentation familiale
- ☐ Habillement
- ☐ Autres

IV.1.1. Si autres, citez

____

IV.2. Pour quelle finalité cultivez-vous le manioc ?

- ◯ Subsistance
- ◯ Vente
- ◯ Les deux

IV.3. Considérant votre capital, la culture du manioc vous procure-t-elle des revenus au cours de l'année?

Les revenus issus de la culture de manioc

- ◯ Oui
- ◯ Non

IV.3.1. Si "OUI" quel est approximativement le montant de revenu issus de la culture de Manioc? [FC]

Combien d'argent obtiens-tu de la culture de manioc par saison?

____

V. SYSTEME DE CULTURE

V.1. Quel type de variété de manioc cultivez-vous?

Le type de variété de manioc que l'agriculteur cultive

- ◯ Variétés Locales
- ◯ Variétés Améliorées
- ◯ Les deux

V.2. Les variétés de manioc que vous cultivez sont de quel goût?

- ◯ Amère
- ◯ Doux
- ◯ Les deux

V.3. Comment cultives-tu le manioc?

- ◯ Monoculture
- ◯ Association
- ◯ Les deux systèmes

V.3.1. Si le manioc est cultivé en association, quels sont les cultures qui sont associées au manioc?
Les cultures associées au manioc

- [ ] Maïs
- [ ] Haricot
- [ ] Patates Douces
- [ ] Sorgho
- [ ] Arachides
- [ ] Riz
- [ ] Oignons
- [ ] Aïl
- [ ] Tomates
- [ ] Autres

Quelles sont les autres cultures non citées parmi les assertions ?

____

V.4. Utilises-tu des fertilisants dans le(s) champs où le manioc est cultivé?
est-ce que l'agriculteur utilise des fertilisants dans le champ où le manioc est cultivé

- ○ Oui
- ○ Non

V.4.1. Si Oui, Quels sont les types de fertilisants utilisés?

- [ ] Fertilisants Organiques
- [ ] Fertilisants minéraux

V.5. Élevez-vous des animaux?

- ○ Oui
- ○ Non

V.5.1. Si Oui, quels animaux élèves-tu?

- [ ] Vache
- [ ] Chèvres
- [ ] Volaille/Basse-cours
- [ ] Porcs
- [ ] Moutons

V.6. Après combien de mois récoltez-vous le manioc de vos champs ?

____

V.7. Quelle quantité de manioc récoltez-vous en moyenne au cours d'une saison si les cultures ne sont pas attaquées par la CBSV?

____

V.9. Quelle est l'unité de production utilisée ?

____

VI.UTILISATION MANIOC

VI.1. Quelles sont les parties de la plante que vous utilisez?
Les parties de la plante qui sont utilisées

- [ ] Tubercules
- [ ] Feuilles
- [ ] Tiges
- [ ] Autres

VI.1.1. Quelles sont les autres parties ?
les autres parties de la plante qui ne sont pas citées parmi les assertions

____

VI.2. Pour quelles finalités les parties de la plante citées sont utilisées?

- [ ] Consommation Humaine
- [ ] Alimentation Animale
- [ ] Mulching
- [ ] Compost
- [ ] Vente
- [ ] Autres

VI.2.1. Quelles sont les autres utilisations

VII. LES BOUTURES

VII.1. D'où obtenez-vous les boutures de manioc que vous cultivez?

- [ ] ONG
- [ ] IITA
- [ ] INERA
- [ ] SENASEM
- [ ] Multiplicateurs des semences
- [ ] Marché
- [ ] Vendeurs d'intrants agricoles
- [ ] Burundi
- [ ] Rwanda
- [ ] Agriculteurs voisins
- [ ] Mon champ personnel
- [ ] Coopérative Agricole
- [ ] Association Paysannes
- [ ] Autres

VII.1.1. Quelles sont les autres sources des boutures non citées parmi les assertions??

VII.2. Quelle distance parcoures-tu pour aller chercher les boutures de manioc?
La distance que l'agriculteur parcours afin d'atteindre le lieu (la source) des boutures de manioc

- ○ Moins de 1Km
- ○ Entre 1 et 5 Km
- ○ Plus de 5Km

VII.3. Par quels moyens obtenez-vous les boutures de manioc?

- [ ] Obtention Gratuite
- [ ] Payement au moyen d'argent
- [ ] Obtention par échange de travail
- [ ] Obtention par échange de récolte
- [ ] Autres

VII.3.1. Quels sont les autres moyens d'obtention des boutures non citées parmi les assertions?

VII.4. Êtes-vous satisfait de la qualité des boutures de manioc que vous cultivez?
La qualité des boutures que cultive l'agriculteur lui satisfont-il

- ○ Oui
- ○ Non

VII.5. Faites-vous confiance en la qualité des boutures que vous cultivez?
Est-ce que l'agriculteur est confiant que la qualité des boutures qu'il cultive est bonne

- ○ Oui
- ○ Non

VII.6. Les boutures que tu obtiens, te suffisent-elles??
Est-ce que les boutures que l'agriculteur parviens à avoir lui sont suffisant

- ○ Oui
- ○ Non

VIII. CONNAISSANCE DES MALADIES DU MANIOC

VIII.1. Connaissez-vous les maladies ou insectes qui attaquent le manioc?

- ○ Oui
- ○ Non

VIII.2. Avez-vous déjà observé des symptômes de maladie de manioc dans un de vos champs?

- ○ Oui
- ○ Non

VIII.2.1. Si "Oui", Connaissez-vous la (les) maladie(s) qui cause(nt) les symptômes observés?
Est-ce que l'agriculteur connaît la maladie qui cause les symptômes observés dans son champ?

- ○ Oui
- ○ Non

VIII.2.2. Si"Oui", quel(s) est(sont) cette(ces) maladies?

- ☐ Mosaïque Africaine de Manioc
- ☐ Striure Brune de Manioc
- ☐ Feu Bactérien du Manioc
- ☐ Autres

Quelles sont les autres causes non citées parmi les assertions?

____

VIII.3. Sur quelles parties de la plante avez-vous observé les symptômes?

- ☐ Feuilles
- ☐ Tiges
- ☐ Tubercules

VIII.4. Savez-vous comment la(les) maladies citées se transmettent?

- ○ Oui
- ○ Non

VIII.4.1. Si "Oui", Quels sont les modes de transmission que vous connaissez?

- ☐ Les boutures
- ☐ La cueillette des feuilles
- ☐ Les outils aratoires
- ☐ Les mouches blanches
- ☐ Autres

Quels sont les autres modes de transmission que vous connaissez?

____

VIII.5. Quelle année avez-vous observé pour la première fois la CBSV dans vos champ ou dans votre Village?

____

VIII.6. Connais-tu comment gérer ou lutter contre ces maladies

- ○ Oui
- ○ Non

VIII.6.1. Si "Oui", comment fais-tu pour lutter ?

- ☐ Enterrer les plantes malades
- ☐ Brûler les plantes malades
- ☐ Utilisation d'insecticides
- ☐ Utilisation des variétés certifiées
- ☐ Utilisation des variétés tolérantes
- ☐ Changer de cultures
- ☐ Abandonner le champ
- ☐ Déraciner complètement la plante
- ☐ Autres

Quelles sont les cultures de remplacement?

- [ ] Patates Douces
- [ ] Maïs
- [ ] Haricots
- [ ] Oignons
- [ ] Sorgho
- [ ] Arachides
- [ ] Légumes
- [ ] Jachère/Pas de culture de remplacement

VIII.6.2. Quels sont les autres moyens de lutte non citées parmi les assertions?

V.8. En cas de maladie de CBSV, Quelle quantité de récolte obtenez-vous de votre champ ?

IX. ACCOMPAGNEMENT DES AGRICULTEURS

IX.1. Avez-vous déjà été visité par un agronome ou un agent de développement dans le cadre de vos activités champêtres?

- ○ Oui
- ○ Non

IX.1.1. Quel genre d'assistance avez-vous reçu de sa part?

- [ ] Visite du champ de manioc
- [ ] Conseils ou formation sur les methodes de gestion de la maladie
- [ ] Conseils ou formation sur les normes de multiplication de manioc
- [ ] Octroi des boutures
- [ ] Autres

IX.1.2. Quels sont les autres types d'assistance non citées parmi les assertions?

IX.2. De quelle institution/organisation est venue cette personne?

- [ ] Je ne sais pas
- [ ] Agronomes ITAPEL (Inspecteurs)
- [ ] INERA
- [ ] IITA
- [ ] Agronomes ONGs
- [ ] Associations paysannes/Coopératives
- [ ] Autres Agriculteurs
- [ ] Etudiants
- [ ] Universités
- [ ] Ecoles agricoles
- [ ] Agents de vulgarisation
- [ ] Autres

Quelles sont les autres provenances non citées parmi les assertions?

X. Quelles sont les principales contraintes que vous rencontrez dans la culture de manioc?

- [ ] Irrégularité des pluies
- [ ] Sécheresse
- [ ] Faible fertilité des champs
- [ ] Longue distance entre le champ et la maison
- [ ] Difficulté de transport de la récolte
- [ ] Main d'oeuvre pour la récolte
- [ ] Manque de champs
- [ ] Petites superficies cultivées
- [ ] Insécurité
- [ ] Manque de boutures saines
- [ ] Problèmes de marché
- [ ] Conflits fonciers
- [ ] Variétés à croissance lente
- [ ] Striure brune de manioc
- [ ] Mosaïque Africaine de Manioc
- [ ] Autres

X.1. Quelles sont les autres contraintes?

____

XI. Que suggérez-vous afin d'améliorer la production de manioc dans votre milieu ?

- [ ] Variétés Résistantes aux maladies
- [ ] Variétés précoces
- [ ] Variétés résistantes à la sécheresse
- [ ] Variétés adaptées aux perturbations climatiques
- [ ] Améliorer le mode de stockage des boutures
- [ ] Implanter les structures de transformation de farine
- [ ] Faciliter le mode de transport des produits
- [ ] Améliorer le marché
- [ ] Autres

XI.1. Quelles sont les autres suggestions qui ne figurent pas parmi les assertions précédentes?

____
