# Supplementary material for: Risk factors associated with cassava brown streak disease dissemination through seed pathways in Eastern D.R. Congo
Source: Front Plant Sci. 2022 Jul 22;13:803980. doi: 10.3389/fpls.2022.803980 (PMC9354974; doi:10.3389/fpls.2022.803980)
Supplement: SUPPLEMENTARY MATERIAL 1 — Questionnaire used for the epidemiological survey in cassava farmer’s fields. [file Data_Sheet_1.zip › Supplementary material/Supplementary Material 3.pdf]

Supplementary material 3

SEED SYSTEM: Multiplicateurs OK

0. Numéro du questionnaire (Commencer par SSM):

____

1. Nom de l'enquêteur

____

I. LOCALISATION DU LIEU DE L'INTERVIEW

I.1. Territoire

- ◯ Uvira
- ◯ Walungu

I.2. Chefferie

- ◯ Bavira
- ◯ Barundi/Plaine de la Ruzizi
- ◯ Bafuliiru

I.3. Village

____

I.6. Coordonnées géographiques du lieu d'interview

latitude (x.y °)

____

longitude (x.y °)

____

altitude (m)

____

précision (m)

____

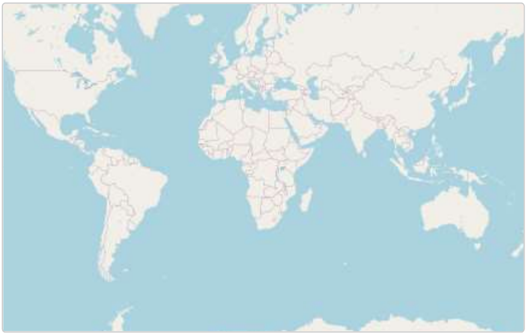

II. IDENTITÉ DU MULTIPLICATEUR DES SEMENCES

II.1. Nom du multiplicateur des boutures

____

II.2. Sexe

- ◯ Homme
- ◯ Femme

II.3. Age

____

II.4. Etat civil

- ◯ Marié
- ◯ Célibataire
- ◯ Divorcé
- ◯ Veuf/Veuve

III. LES BOUTURES DE MANIOC

III.1. Quels sont les types de variétés que vous multipliez souvent?

- ◯ Variétés améliorées
- ◯ Variétés locales
- ◯ Les deux

III.2. Où obtenez-vous les boutures de ces variétés que vous Multipliez?

- [ ] ONGs
- [ ] IITA
- [ ] INERA
- [ ] SENASEM
- [ ] Autres multiplicateurs des semences
- [ ] Marché
- [ ] Vendeurs d'intrants agricoles
- [ ] Rwanda
- [ ] Burundi
- [ ] Agriculteurs voisins
- [ ] Mon propre champ
- [ ] Association paysanne/coopérative
- [ ] Autres

III.2.1. Quelles sont les autres sources non citées?

III.3. Quelle distance parcoures-tu pour aller chercher les boutures à Multiplier?

- ◯ <5Km
- ◯ 1-5Km
- ◯ >5Km

III.4. Par quel(s) moyens vous obtenez les boutures à Multiplier?

- [ ] Obtention gratuite
- [ ] Paiement au moyen d'argent
- [ ] Obtention par échange de travail
- [ ] Obtention par échange de récolte
- [ ] Autres

III.4.1. Quels sont les autres moyens d'obtention des boutures qui ne sont pas citées parmi les assertions?

III.5. Avez-vous des critères pour choisir les types de variétés à multiplier?

- ◯ Oui
- ◯ Non

III.5.1. Si "OUI", quels sont ces critères?

- [ ] Durée du cycle de croissance
- [ ] Exigences climatiques (Sécheresse, irrégularité pluies)
- [ ] Exigences de sol
- [ ] Résilience aux attaques d'insectes
- [ ] Résilience aux attaques des maladies
- [ ] Caractéristiques nutritionnelles
- [ ] Caractéristiques gustatives
- [ ] Aspects culinaires
- [ ] Aptitudes à la transformation
- [ ] Autres

III.5.2. Quels sont les autres critères qui ne figurent pas parmi les assertions?

III.6. Utilisez-vous des critères pour sélectionner les boutures à multiplier?

- ◯ Oui
- ◯ Non

III.6.1. Si "OUI", quels sont ces critères?

- [ ] Couleur
- [ ] Grosseur/épaisseur/grandeur
- [ ] Age
- [ ] Vigueur
- [ ] Taux de germination
- [ ] Productivité
- [ ] Homogénéité (pour l'arrivée à maturité ou pour des signes distinctifs particuliers)
- [ ] Heterogénéité (lors de l'attaque des maladies ou face à la variabilité des conditions climatiques)
- [ ] Présence des symptômes des maladies
- [ ] Absence des symptômes des maladies
- [ ] Absence d'insectes ou d'organismes pathogènes
- [ ] Autres

III.6.2. Quels sont les autres critères qui ne figurent pas parmi les assertions?

____

III.7. Êtes-vous satisfait de la qualité des boutures de manioc que vous multipliez?

- ○ Oui
- ○ Non

III.8. Faites-vous confiance en la qualité des boutures que vous multipliez?

- ○ Oui
- ○ Non

III.9. Quels sont tes principaux clients/acheteurs des boutures de manioc?

- [ ] Les agriculteurs
- [ ] ONGs
- [ ] Les autres multiplicateurs des boutures
- [ ] INERA
- [ ] IITA
- [ ] SENASEM
- [ ] ITAPEL
- [ ] Universités/Ecoles agricoles
- [ ] Agrodealers
- [ ] Fermes agricoles
- [ ] Autres

II.9.1. Quelles sont les autres Clients/acheteurs non cités parmi les assertions?

____

III.10. Les boutures que tu multiplies, satisfont-elles la demande?

- ○ Oui
- ○ Non

III.11. Quels sont les types de variétés qui sont préférées par vos clients?

- ○ Locales
- ○ Améliorées
- ○ Les deux

IV. CONNAISSANCE DES MALADIES DU MANIOC

IV.1. Connaissez-vous les maladies ou insectes qui attaquent le manioc?

- ○ Oui
- ○ Non

IV.2. Avez-vous déjà observé des symptômes de maladie de manioc dans un de vos champs de Multiplication?

- ○ Oui
- ○ Non

IV.2.1. Si "Oui", Connaissez-vous la (les) maladie(s) qui cause(nt) les symptômes observés?

- Oui
- Non

IV.2.2. Si"Oui", quel(s) est(sont) cette(ces) maladies?

- Mosaïque Africaine de Manioc
- Striure Brune de Manioc
- Feu bactérien du manioc
- Autres

IV.2.3. Quelles sont les autres causes non citées parmi les assertions?

IV.3. Sur quelles parties de la plante avez-vous observé les symptômes?

- Feuilles
- Tiges
- Racines/Tubercules

IV.4. Savez-vous comment la(les) maladies citées se transmettent?

- Oui
- Non

IV.4.1. Si "Oui", Quels sont les modes de transmission que vous connaissez?

- Les boutures
- La cueillette des feuilles
- Les outils aratoires
- Les mouches blanches
- Autres

IV.4.1.1. Quels sont les autres modes de transmission non citées parmi les assertions?

IV.5. Connais-tu comment gérer ou lutter contre ces maladies

- Oui
- Non

IV.5.1. Si "Oui", comment fais-tu pour lutter ?

- Enterrer les plantes
- Brûler les plantes
- Utilisation d'insecticides
- Utilisation des variétés certifiées
- Utilisation des variétés tolérantes
- Changer les cultures
- Abandonner le champ/le laisser en jachère
- Déraciner complètement la plante
- Autres

IV.5.2. Quelles sont les cultures de remplacement?

- Maïs
- Haricot
- Patate Douce
- Sorgho
- Arachide
- Aïl
- Oignons
- Tomates
- Riz
- Autres

IV.5.2. Quels sont les autres moyens de lutte non citées parmi les assertions?

IV.5.2.1. Quelles sont les autres cultures de remplacement non citées parmi les assertions??

____

V. SYSTÈME DE CULTURE DU CHAMP DE MULTIPLICATION

V.3. Comment cultives-tu le manioc destiné à la multiplication des boutures??

- ◯ Association culturale
- ◯ Monoculture
- ◯ Les deux

V.3.1. Quelles sont les cultures associées au manioc en multiplication?

- ☐ Maïs
- ☐ Haricot
- ☐ Patates douces
- ☐ Sorgho
- ☐ Arachides
- ☐ Riz
- ☐ Oignons
- ☐ Aïl
- ☐ Tomates
- ☐ Autres

V.3.2. Quelles sont les autres cultures non citées parmi les assertions ?

____

V.4. Utilisez-vous des fertilisants dans votre champ de multiplication des boutures?

- ◯ Oui
- ◯ Non

V.4.1. Si Oui, Quels sont les types de fertilisants utilisés?

- ◯ Fertilisants minéraux
- ◯ Fertilisants organiques
- ◯ Les deux

V.5. Élevez-vous des animaux?

- ◯ Oui
- ◯ Non

V.5.1. Si Oui, quels animaux élèves-tu?

- ☐ Chèvres
- ☐ Vaches
- ☐ Porcs
- ☐ Moutons
- ☐ Basse-cours
- ☐ Autres

V.5.2. Quels sont les autres animaux non cités parmi les assertions précédentes?

____

V.6. Après combien de mois commencez-vous à récolter les boutures pour la vente ?

____

V.7. Quelle quantité de boutures récoltez-vous en moyenne au cours d'une saison ?

____

V.7.1. Quelle unité de mesure pour les boutures?

____

V.8. Quelle quantité de manioc récoltez-vous en moyenne dans ce champ de multiplication au cours d'une saison?

____

V.8.1. Quelle unité de mesure pour les tubercules?

____

VI. ACCOMPAGNEMENT DES MULTIPLICATEURS DES BOUTURES

VI.1. Etes-vous suivi ou accompagné par des agents du service public dans vos activités de multiplication des boutures?

- ◯ Oui
- ◯ Non

VI.1.1. Quel genre d'assistance avez-vous reçu?

- ☐ Visite du champ de manioc
- ☐ Conseils ou formation sur les méthodes de gestion de la maladie
- ☐ Conseils ou formation sur les normes de multiplication de manioc
- ☐ Octroi des boutures
- ☐ Autres

VI.1.2. Quels sont les autres types d'assistance non citées parmi les assertions ?

____

VI.2. Quels sont les services/intervenants qui vous ont suivi ou accompagné dans vos activités de multiplication des boutures de manioc?

- ☐ Agronomes IPAPEL (ou inspecteurs agricoles)
- ☐ INERA
- ☐ IITA
- ☐ Agronomes ONGs
- ☐ Associations paysannes/coopératives
- ☐ Autres multiplicateurs des semences
- ☐ Universités
- ☐ Ecoles agricoles
- ☐ Agents de vulgarisation
- ☐ Autres

VI.2.1. Quels sont les "Autres" services/Intervenants non cités parmi les assertions précédentes qui vous ont suivis?

____

VI.3. Connaissez-vous les conditions et normes requis pour être multiplicateur agréé des boutures de manioc?

- ◯ Oui
- ◯ Non

VI.4. Les boutures que vous vendez, sont-elles certifiées/autorisées?

- ◯ Oui
- ◯ Non

V.II. BUSINESS DES BOUTURES

VII.1. En quoi est-ce que l'activité de multiplication des boutures de manioc améliore ta vie et celle de ton ménage?

- ☐ Payement des frais scolaires
- ☐ Soins de santé
- ☐ Alimentation de la famille
- ☐ Habillement
- ☐ Autres

VII.1.1. Quels sont les autres réponses qui ne figurent pas parmi les assertions précédentes?

____

VII.2. Considérant votre capital, l'activité de multiplication des boutures de manioc vous procure-t-elle des revenus au cours de l'année?

Est-ce-que l'activité de multiplication des boutures génère des revenus ?

- ◯ Oui
- ◯ Non

VII.2.1. Pouvez-vous estimer le montant de revenu que l'activité de multiplication des boutures de manioc vous procure au courant de l'année? (n'oublie pas de mentionner la monnaie!!!!!)

____

VII.3. A part les Boutures, quels sont les autres produits issus du champ de multiplication des boutures que vous utilisez ?

- [ ] Les feuilles
- [ ] Les tubercules/racines

VIII. Quelles sont les principales contraintes que vous rencontrez dans votre activité de multiplication des boutures de manioc?

- [ ] Irrégularité des pluies
- [ ] Sécheresse
- [ ] Faible fertilité des champs
- [ ] Long trajet vers les champs
- [ ] Transport de la récolte
- [ ] Main d'oeuvre pour la récolte
- [ ] Manque des champs
- [ ] Petites superficies cultivées
- [ ] Insécurité
- [ ] Manque des boutures saines
- [ ] Problèmes de marché/Business des boutures
- [ ] Divagation des bêtes
- [ ] Conflits fonciers
- [ ] Longue durée de croissance
- [ ] Striure Brune de manioc
- [ ] Mosaïque Africaine de Manioc
- [ ] Autres maladies et ravageurs
- [ ] Autres

VIII.1. Quelles sont les autres contraintes non citées parmi les assertions ?

____

IX. Que suggérez-vous afin d'améliorer la multiplication des boutures de manioc?

- [ ] Variétés précoces
- [ ] Variétés résistantes à la sécheresse
- [ ] Variétés adaptées aux perturbations climatiques
- [ ] Améliorer le mode de stockage des boutures
- [ ] Implanter les structures de transformation
- [ ] Faciliter le moyen de transport
- [ ] Améliorer le marché des boutures
- [ ] Autres

IX.1. Quelles sont les autres suggestions qui ne figurent pas parmi les assertions précédentes?

____

X. Selon vous, qu'est-ce qui peut être fait afin d'améliorer la fourniture des boutures "saines" de manioc dans votre milieu?

____
