# Supplementary material for: Risk factors associated with cassava brown streak disease dissemination through seed pathways in Eastern D.R. Congo
Source: Front Plant Sci. 2022 Jul 22;13:803980. doi: 10.3389/fpls.2022.803980 (PMC9354974; doi:10.3389/fpls.2022.803980)
Supplement: SUPPLEMENTARY MATERIAL 1 — Questionnaire used for the epidemiological survey in cassava farmer’s fields. [file Data_Sheet_1.zip › Supplementary material/Supplementary Table 1.docx]

**Supplementary Table 1**. Proportion of fields grown by different types of cutting pathways and means employed by farmers to obtain cuttings across clusters.

| **Characteristics** | **Cluster 1 [**80]*^1^* | **Cluster 2 [**85] | **Cluster 3 [**81]^2^ | **Overall [**246] | **p-value** |
| --- | --- | --- | --- | --- | --- |
| ***Cutting pathways*** | |  |  |  | 0.04 |
| Farmers (F) | 48% _[26]_ | 32% _[17]_ | 20% _[11]_ | 100% _[54]_ |  |
| F+Cooperatives (C) | 30% _[31]_ | 27% _[28]_ | 43% _[44]_ | 100% _[103]_ |  |
| F+C+Market | 18% _[5]_ | 64% _[18]_ | 18% _[5]_ | 100% _[28]_ |  |
| F+C+Seed Multipliers | 31% _[16]_ | 39% _[20]_ | 29% _[15]_ | 100% _[51]_ |  |
| F+ Neighbour Country | 50% _[2]_ | 50% _[2]_ | - | 100% _[4]_ |  |
| ***Means of cutting obtention*** | |  |  |  | 0.001 |
| Free | 50% _[40]_ | 28% _[24]_ | 56% _[45]_ | 44% _[109]_ |  |
| +Money | 28% _[22]_ | 55% _[47]_ | 41% _[33]_ | 42% _[102]_ |  |
| +Work/Yield | 23% _[18]_ | 17% _[14]_ | 4% _[3]_ | 14% _[35]_ |  |
| *^1^*[n]: Numbers in brackets represents the number of fields grown by cuttings obtained from the corresponding pathways/pathways | | | | |  |
| ^2^: No data on the pathways used to obtain cuttings in 6 fields of the cluster 3 could be noticed. | | | | |  |
| -: The modality related to that cutting pathway was absent in the corresponding cluster. | | | | |  |
| *^2^*Pearson's Chi-squared test | | | | |  |
