# Supplementary material for: Risk factors associated with cassava brown streak disease dissemination through seed pathways in Eastern D.R. Congo
Source: Front Plant Sci. 2022 Jul 22;13:803980. doi: 10.3389/fpls.2022.803980 (PMC9354974; doi:10.3389/fpls.2022.803980)
Supplement: SUPPLEMENTARY MATERIAL 1 — Questionnaire used for the epidemiological survey in cassava farmer’s fields. [file Data_Sheet_1.zip › Supplementary material/Supplementary Table 2.docx]

**Supplementary Table 2**. Number of fields grown by improved as well as local cassava varieties identified in each cluster

| **Improved varieties** | | | | |
| --- | --- | --- | --- | --- |
| ***Local names*** | ***Cluster 1*** | ***Cluster 2*** | ***Cluster 3*** | ***Overall*** |
| Dorothea | 7 | 7 | 29 | 43 |
| V8 | 24 | 1 | 14 | 39 |
| Sawa sawa | 3 | 24 | 10 | 37 |
| Magouringware | 8 | 8 | 11 | 27 |
| Mabwaki | 1 | 7 | 3 | 11 |
| Nabwigoma | 7 | 0 | 1 | 8 |
| Bwika | 3 | 3 | 0 | 6 |
| Kantintima | 0 | 5 | 1 | 6 |
| Namuzungu | 4 | 1 | 1 | 6 |
| Rava | 0 | 3 | 2 | 5 |
| Nabwilalanga | 1 | 0 | 3 | 4 |
| Liyayi | 0 | 0 | 3 | 3 |
| Mayombe | 3 | 0 | 0 | 3 |
| Mvuama | 0 | 0 | 3 | 3 |
| Nakahegere | 1 | 0 | 2 | 3 |
| Mukombe | 0 | 2 | 0 | 2 |
| Naluvuzi | 0 | 0 | 2 | 2 |
| Ndunda | 0 | 2 | 0 | 2 |
| Butamu | 0 | 1 | 0 | 1 |
| Kihonya | 0 | 0 | 1 | 1 |
| Mabwilalanga | 0 | 0 | 1 | 1 |
| Migera | 1 | 0 | 0 | 1 |
| Nakaronda | 0 | 1 | 0 | 1 |
| **Local Varieties** | | | | |
| ***Local names*** | ***Cluster 1*** | ***Cluster 2*** | ***Cluster 3*** | ***Overall*** |
| Nahunde | 6 | 7 | 2 | 15 |
| Nakarasi | 1 | 4 | 2 | 7 |
| Kahungu | 1 | 0 | 2 | 3 |
