# Supplementary material for: Risk factors associated with cassava brown streak disease dissemination through seed pathways in Eastern D.R. Congo
Source: Front Plant Sci. 2022 Jul 22;13:803980. doi: 10.3389/fpls.2022.803980 (PMC9354974; doi:10.3389/fpls.2022.803980)
Supplement: SUPPLEMENTARY MATERIAL 1 — Questionnaire used for the epidemiological survey in cassava farmer’s fields. [file Data_Sheet_1.zip › Supplementary material/Supplementary Table 3.docx]

**Supplementary Table 3**. Proportion of healthy and infected fields for each type of infection according to the channels used to obtain cutting

| **Characteristic** | **CBSV INFECTION** | | | **UCBSV INFECTION** | | | **CBSV+UCBSV INFECTION** | | | **OVERALL (Infected vs healthy)** | | |
| --- | --- | --- | --- | --- | --- | --- | --- | --- | --- | --- | --- | --- |
|  | **Absence (**N=218*^1^***)** | **Presence** (N=28*^1^***)** | **Overall**, N=246*^1^* | **Absence (N=212***^1^***)** | **Presence (**N=34*^1^***)** | **Overall**, N=246*^1^* | **Absence (N=228***^1^***)** | **Presence** (N=18*^1^***)** | **Overall**, N=246*^1^* | **Presence (**N=80*^1^***)** | **Absence (**N=166*^1^***)** | **Overall**, N=246*^1^* |
| ***Cutting channels*** |  |  |  |  |  |  |  |  |  |  |  |  |
| Farmers (F) | 87% [47] | 13% [7] | 100% [54] | 85% [46] | 15% [8] | 100% [54] | 87% [47] | 13% [7] | 100% [54] | 41% [22] | 59% [32] | 100% [54] |
| F+Cooperatives (C) | 90% [93] | 10% [10] | 100% [103] | 82% [84] | 18% [19] | 100% [103] | 94% [97] | 6% [6] | 100% [103] | 34% [35] | 66% [68] | 100% [103] |
| F+C+Market (M) | 86% [24] | 14% [4] | 100% [28] | 89% [25] | 11% [3] | 100% [28] | 89% [25] | 11% [3] | 100% [28] | 36% [10] | 64% [18] | 100% [28] |
| F+C+ Seeds Multipliers | 88% [45] | 12% [6] | 100% [51] | 94% [48] | 6% [3] | 100% [51] | 96% [49] | 4% [2] | 100% [51] | 22% [11] | 78% [40] | 100% [51] |
| F+Neighbor Countries | 100% [4] | - | 100% [4] | 100% [4] | - | 100% [4] | 100% [4] | - | 100% [4] | - | 100% [4] | 100% [4] |
| **p-value2** | **0.9** | | | **0.3** | | | **0.4** | | | **0.2** | | |
| *Missing values* | 5 | 1 | 6 | 5 | 1 | 6 | 6 | 0 | 6 | 2 | 4 | 6 |
| *^1^*% [n] | | | | | | | | | | | | |
| *^2^*Fisher's exact test | | | | | | | | | | | | |
