# Supplementary figures and images for: Risk factors associated with cassava brown streak disease dissemination through seed pathways in Eastern D.R. Congo
Source: Front Plant Sci. 2022 Jul 22;13:803980. doi: 10.3389/fpls.2022.803980 (PMC9354974; doi:10.3389/fpls.2022.803980)

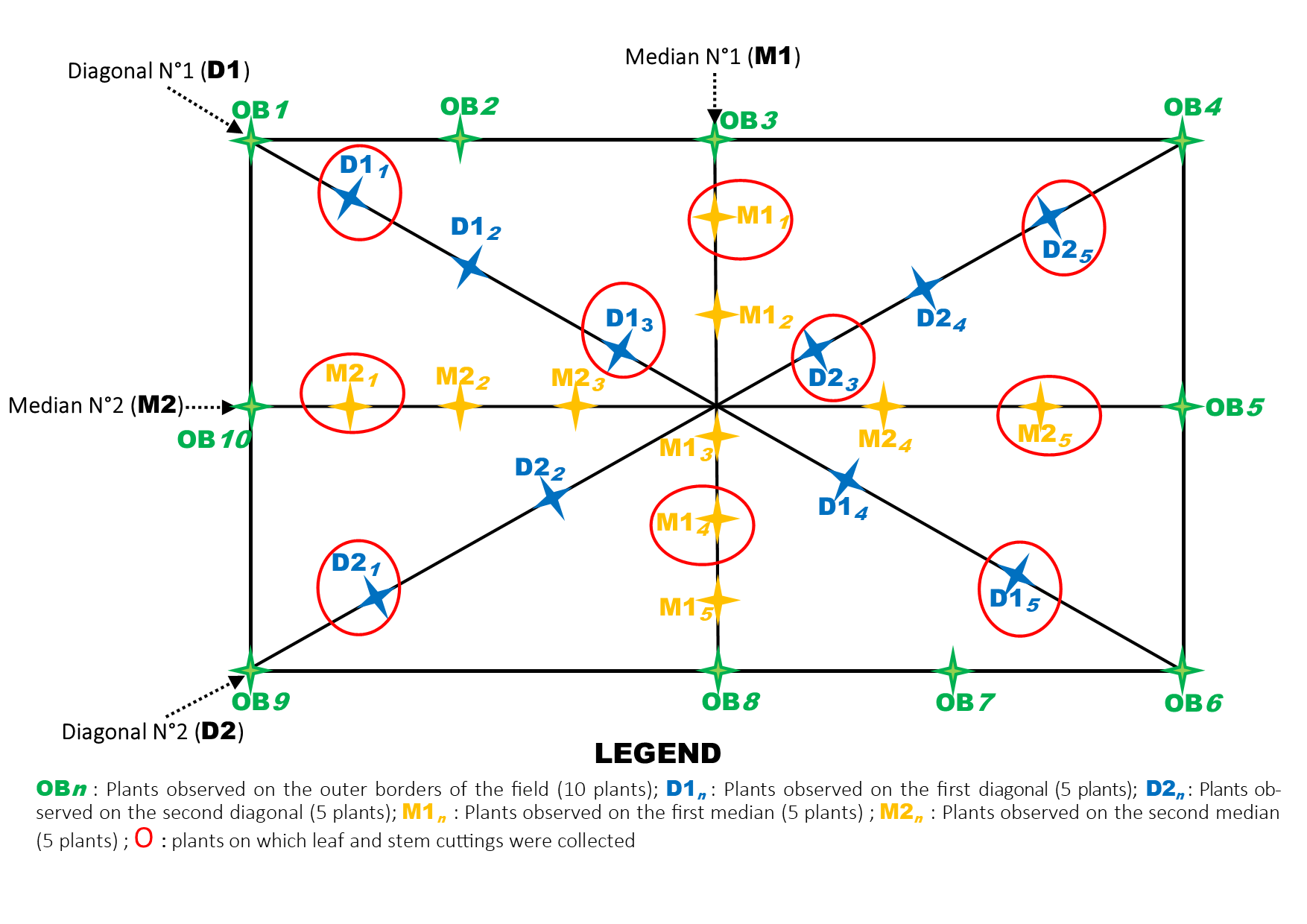

Supplement: SUPPLEMENTARY MATERIAL 1 — Questionnaire used for the epidemiological survey in cassava farmer’s fields. [file Data_Sheet_1.zip › Supplementary material/Supplementary Figure 1.TIF]

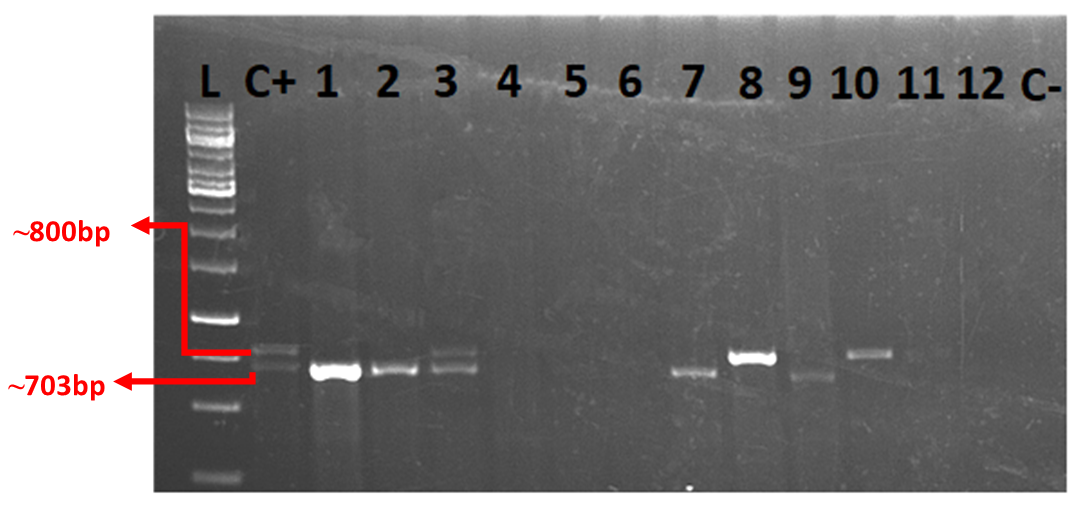

Supplement: SUPPLEMENTARY MATERIAL 1 — Questionnaire used for the epidemiological survey in cassava farmer’s fields. [file Data_Sheet_1.zip › Supplementary material/Supplementary Figure 2.TIF]

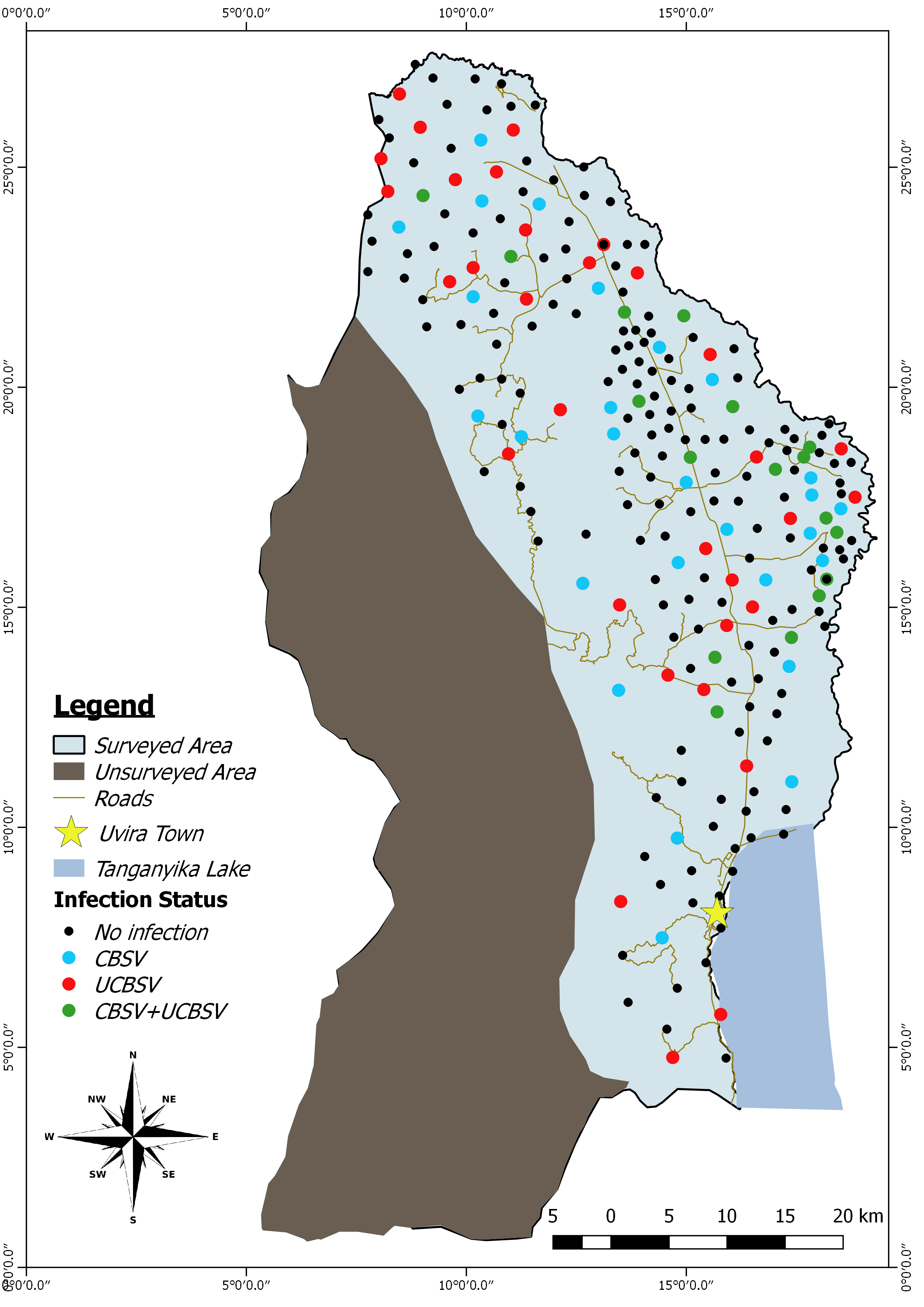

Supplement: SUPPLEMENTARY MATERIAL 1 — Questionnaire used for the epidemiological survey in cassava farmer’s fields. [file Data_Sheet_1.zip › Supplementary material/Supplementary Figure 3.TIF]
